# Tuning Ambipolarity of the Conjugated Polymer Channel Layers of Floating‐Gate Free Transistors: From Volatile Memories to Artificial Synapses

**DOI:** 10.1002/advs.202203025

**Published:** 2022-08-19

**Authors:** Yu‐Ting Yang, Ying‐Sheng Wu, Waner He, Hsin‐Chiao Tien, Wei‐Chen Yang, Tsuyoshi Michinobu, Wen‐Chang Chen, Wen‐Ya Lee, Chu‐Chen Chueh

**Affiliations:** ^1^ Department of Chemical Engineering National Taiwan University Taipei 10617 Taiwan; ^2^ Department of Materials Science and Engineering Tokyo Institute of Technology 2‐12‐1 Ookayama, Meguro‐ku Tokyo 152‐8552 Japan; ^3^ Research and Development Center for Smart Textile Technology and Department of Chemical Engineering and Biotechnology National Taipei University of Technology Taipei 106 Taiwan; ^4^ Advanced Research Center of Green Materials Science and Technology National Taiwan University Taipei 10617 Taiwan

**Keywords:** ambipolarity, conjugated polymers, floating‐gate free transistors, synaptic transistors, transistor memory

## Abstract

Three‐terminal synaptic transistor has drawn significant research interests for neuromorphic computation due to its advantage of facile device integrability. Lately, bulk‐heterojunction‐based synaptic transistors with bipolar modulation are proposed to exempt the use of an additional floating gate. However, the actual correlation between the channel's ambipolarity, memory characteristic, and synaptic behavior for a floating‐gate free transistor has not been investigated yet. Herein, by studying five diketopyrrolopyrrole–benzotriazole dual‐acceptor random conjugated polymers, a clear correlation among the hole/electron ratio, the memory retention characteristic, and the synaptic behavior for the polymer channel layer in a floating‐gate free transistor is described. It reveals that the polymers with balanced ambipolarity possess better charge trapping capabilities and larger memory windows; however, the high ambipolarity results in higher volatility of the memory characteristics, namely poor memory retention capability. In contrast, the polymer with a reduced ambipolarity possesses an enhanced memory retention capability despite showing a reduced memory window. It is further manifested that this enhanced charge retention capability enables the device to present artificial synaptic characteristics. The results highlight the importance of the channel's ambipolarity of floating‐gate free transistors on the resultant volatile memory characteristics and synaptic behaviors.

## Graphical abstract

A clear correlation among the hole/electron mobility (*µ*_h_/*µ*_e_) ratio, the memory retention characteristic, and the synaptic behavior for the polymer channel layer in a floating‐gate free transistor is demonstrated. It shows that, compared to the device with a high ambipolarity (*µ*_h_/*µ*_e_ ratio: 1), the device with a low ambipolarity (*µ*_h_/*µ*_e_ ratio: 10) can exhibit an enhanced charge retention capability but possessing a reduced memory window. However, such enhanced charge retention capability enables the device to present the artificial synaptic characteristics.

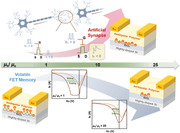

## 1 Introduction

Traditional digital computing systems have encountered the so‐called “von Neumann bottleneck,” which is an inefficient information processing due to the separation of memory modules and processing units.^[^ ^1^, ^2^ ^]^ To counter this, researchers attempt to develop a new computing system by imitating the human brain. The human brain is a highly efficient biological computing system that can perform complex calculations through the constituent neurons.^[^ ^3^, ^4^ ^]^ In this system, synapses are the basic units responsible for the signal transmission between neurons, equivalent to the data processing in the traditional computing system.^[^ ^5^ ^]^ Hence, the development of artificial synapses gained explosive growth in recent years because they help build the next generation brain‐like computers with very high data processing efficiencies and low energy consumption.^[^ ^6^ ^]^

To date, various types of synaptic devices have been developed, including phase‐change transistors,^[^ ^7^, ^8^ ^]^ memristors,^[^ ^9^, ^10^ ^]^ and three‐terminal transistor memories.^[^ ^11^, ^12^ ^]^ Among them, the three‐terminal transistor memory shows a specific potential for neuromorphic applications because its device structure affords reduced crosstalk between each unit and is easily integrated with other circuits. In principle, the gate stimulus for a three‐terminal transistor can serve as the presynaptic input to trigger/reduce the drain current, thereby generating the excitatory postsynaptic current (EPSC)/the inhibitory postsynaptic current (IPSC).^[^ ^13^ ^]^ Notably, different than the conventional transistors, concurrent charge transport and storage must be achieved in the synaptic transistors to trigger EPSC/IPSC and other synaptic functions, like the paired‐pulse facilitation (PPF), potentiation/depression (LTP/LTD), spike‐voltage‐dependent plasticity (SVDP), and spike‐rate‐dependent plasticity (SRDP), to successfully mimic the artificial synapses.^[^ ^13^, ^14^ ^]^ Therefore, an additional chargeable dielectric layer, including floating gate, ferroelectrics, and ionic gels, is commonly implanted into a regular transistor device to fulfill the memory and synaptic characteristics.^[^ ^15^, ^16^, ^17^, ^18^, ^19^, ^20^, ^21^, ^22^, ^23^ ^]^ For example, Guo et al. reported a synaptic transistor memory based on a p‐type polymer channel layer by introducing a perovskite quantum dot‐based floating gate.^[^ ^18^ ^]^

Nevertheless, the synaptic transistors with the additional chargeable dielectric layer still suffer from several challenges. One of them lies in their multilayer manufacturing processes, where the deposition of the chargeable dielectric layer will not only affect the formation/quality of the atop channel layer but also add the production cost. To address this issue, researchers propose a new type of synaptic transistor that adopts a bulk‐heterojunction p‐/n‐type semiconductor blend to realize the concurrent charge transport and storage behaviors in the active channel layer to exempt the use of a floating gate.^[^ ^24^, ^25^, ^26^, ^27^, ^28^, ^29^, ^30^, ^31^, ^32^ ^]^ For instance, Guo et al. showed a novel synaptic transistor by doping an n‐type conjugated polymer into a p‐type polymer channel layer and investigated the doping effect on the resultant synaptic behaviors.^[^ ^25^ ^]^ In the meanwhile, Lee et al. found that doping a p‐type polymer channel layer with an ionic salt can make a regular transistor device possess synaptic characteristics.^[^ ^33^ ^]^ Such bulk‐heterojunction design reveals that the polarity modulation for an active channel imposes a critical influence on the resultant memory and synaptic characteristics. Similar phenomena were also observed in the donor–acceptor (D–A) copolymer design. The coexisting donor and acceptor moieties can afford concurrent charge transport and storage to endow the derived transistor with conspicuous memory or synaptic characteristics without using a floating gate.^[^ ^33^, ^34^, ^35^ ^]^ Despite these demonstrated results, the actual correlation among the polymer's ambipolarity, the memory characteristic, and the synaptic behavior has not yet been investigated in the literature. Furthermore, the influence of the degree of unbalance between hole and electron mobilities on the resultant performance is also not clarified.

Over the past decades, many ambipolar conjugated polymers have been synthesized and used in various kinds of device applications.^[^ ^36^, ^37^, ^38^ ^]^ Among which, diketopyrrolopyrrole (DPP)‐based D–A copolymers attracted particular research attention for transistor applications due to their high charge transport properties.^[^ ^39^, ^40^, ^41^ ^]^ On the other hand, the dual‐acceptor configuration of conjugated polymers has recently drawn wide research interests because it promotes the charge transport by affording a relatively low‐lying highest occupied molecular orbital (HOMO) and lowest unoccupied molecular orbital (LUMO) levels.^[^ ^42^ ^]^ Hence, we are interested in designing a series of DPP‐based dual‐acceptor conjugated polymers with varying degrees of ambipolarity and investigate the influence of the hole/electron (*µ*_h_/*µ*_e_) mobility ratio on the resultant memory and synaptic characteristics.

**Figure 1:**
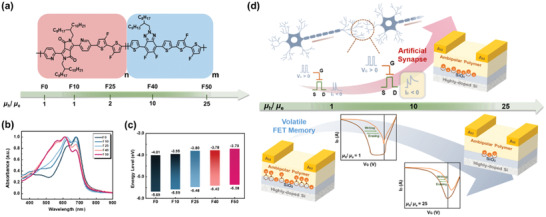
a) Chemical structures, b) UV–vis absorption spectra, and c) energy levels of the target DPP‐based dual‐acceptor random polymers, where *n*:*m* = 100:0 (F0), 90:10 (F10), 75:25 (F25), 60:40 (F40), and 50:50 (F50). d) The correlation between the hole/electron mobility ratio of the polymer channel and the resultant memory, synaptic characteristics.

In this study, we synthesize five dual‐acceptor conjugated polymers (F0, F10, F25, F40, and F50) through the random copolymerization of the DPP unit and the benzotriazole unit at varied molar ratios (**Figure** **1**a). Transistors based on these polymers show gradually increasing *µ*_h_/*µ*_e_ mobility ratios from F0 to F50. The balanced *µ*_h_/*µ*_e_ mobility of F0 is gradually converted to a hole‐dominated carrier transport of F50 as the content of the benzotriazole unit increases. These devices also demonstrate memory characteristics without adding an extra chargeable dielectric layer or a floating gate. The different memory window and charge retention capabilities of these devices correlate with the varying ambipolarity of the polymers. The diminishing ambipolarity from F0 to F50 leads to a gradually reduced memory window of the derived transistor memory but with gradually increasing memory retention capability, especially for F40 with a *µ*_h_/*µ*_e_ mobility ratio of 10. Surprisingly, the best transient storage capability of F40 endows its device with the most conspicuous artificial synaptic characteristics, such as PPF, SVDP, and SRDP, among the five polymers. This study reveals an unexpected compromise between the volatile memory characteristics and the artificial synaptic characteristics for a floating‐gate free transistor memory. This dependence on polymer's ambipolarity (Figure 1d) provides a clear design principle for the organic floating‐gate free synaptic transistors.

## 2 Results and Discussion

### 2.1 Syntheses and Characterizations of the Dual‐Acceptor Conjugated Polymers

A series of pyridine‐flanked DPP‐based dual‐acceptor conjugated polymers was herein synthesized through the random copolymerization of a pyridine‐flanked DPP monomer (M1) with a fluorinated benzotriazole monomer (M2). M1 was synthesized according to the procedures reported in the literature,^[^ ^40^ ^]^ and the ^1^H NMR spectrum presented in Figure S1, Supporting Information, confirms its successful preparation. By controlling the molar ratios between M1 and M2 and coupling with a (3,3′‐difluoro‐[2,2′‐bithiophene]‐5,5′‐diyl)bis(trimethylstannane) (2T‐F) bridge moiety, five target polymers (F0, F10, F25, F40, and F50) containing 0%, 10%, 25%, 40%, and 50% of M2 were prepared. The polymerization details are described in the Supporting Information. Notably, increasing the M2 content to >50% led to the low molecular weight of the prepared polymer because of the substitution of the carbon side chains with fluoride in M2.

The ^1^H NMR spectra in Figures S2–S6, Supporting Information, and the corresponding elemental analyses confirm the successful polymerization of F0–F50. By analyzing the elemental composition of each polymer, it is obvious that the sulfur and nitrogen content increased, and the hydrogen content decreased as increasing the content of M2. Attributed to the attached branch side chains, F0–F50 possess decent solubility in common organic solvents, like tetrahydrofuran (THF), chloroform, and toluene. Their molecular weights measured by a THF‐eluted size exclusion chromatography (SEC) are summarized in Table S1, Supporting Information. The number‐average molecular weight (*M*_n_) values are in the range of 24.2 to 37.9 kDa with *Đ* values between 2 and 3. Their thermal stability measured by thermogravimetric analysis (TGA) is presented in Figure S7a, Supporting Information. All of them exhibited a high thermal decomposition temperature (*T*_d,5%_) of >300 °C, revealing a great thermal stability. Notably, the thermal stability of the polymers slightly increases as the content of M2 increases (Table S1, Supporting Information). It is due to the better thermal resistance of the M2 unit than the DPP unit. Figure S7b, Supporting Information, and Figure 1b, respectively, show the solution (in chlorobenzene) and film ultraviolet−visible (UV−vis) absorption spectra of F0–F50. As shown, the solution and film spectra are similar, and all polymers displayed a similar onset wavelength despite the varying content of M2. However, as increasing the content of M2, the maximum absorption peak was gradually blue‐shifted from 675 to 590 nm, owing to its absorption feature. Meanwhile, the absorption peak at ≈675 nm associated with the short‐range aggregation was gradually decreased. This result suggests that the random structure disrupts the packing between the polymer chains. Another advantage to randomize the regular polymer is to fine‐tune the energy levels without largely altering the bandgap. To confirm this, cyclic voltammetry (CV) was conducted, and the details for the measurement and results are listed in Table S1, Supporting Information. As shown, all polymers indeed possess a similar bandgap (1.64–1.68 eV), but their corresponding HOMO/LUMO levels were gradually upward shifted from 5.69/4.01 eV (F0) to 5.38/3.70 eV (F50) (Figure 1c). Such fine‐tuned energy levels impose a non‐trivial influence on the polymer's polarity which will be discussed later.

### 2.2 Typical OFET Performance

**Figure 2:**
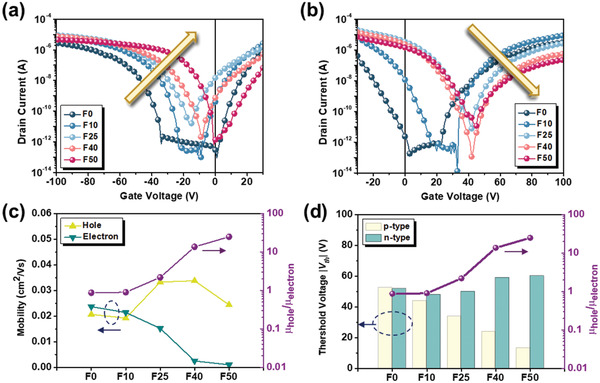
a) p‐Type transfer characteristics of F0–F50 transistor devices, where the gate voltage (*V*_GS_) was swept from +30 to −100 V at a fixed drain voltage (*V*_DS_) of −100 V and b) the corresponding n‐type transfer characteristics measured at a *V*_DS_ of 100 V with *V*_GS_ swept from −30 to +100 V. Summary of c) *µ*_h_, *µ*_e_, and the *µ*_h_/*µ*_e_ ratio and d) the threshold voltages (*V*_th_s) of F0–F50 transistor devices measured in a N_2_‐filled glove box.

The charge transport properties of F0–F50 were examined in a bottom gate/top contact transistor device. Device fabrication details are described in the Experimental Section. **Figure** **2**a presents the p‐type transfer curves for the F0–F50 devices measured at a fixed drain voltage (*V*_DS_) of −100 V with the gate voltage (*V*_GS_) swept from +30 to −100 V. As can be seen, from F0 to F50, the transfer curve was gradually shifted to the positive voltage direction and associated with an increased on current. This change manifests the gradually enhanced p‐type characteristic from F0 to F50. This was also supported by their corresponding p‐type output curves (Figure S8, Supporting Information). Table S2, Supporting Information, summarizes the performance of the F0–F50 devices, and Figures 2c and 2d, respectively, depict the changes in *µ*_h_ values and the threshold voltages (*V*_th_s) of these devices. The gradually increasing *µ*_h_ value from F0 to F40 can be attributed to the increased HOMO level (Figure 1c), which reduces the hole injection barrier with the Au electrode (−5.0 eV).^[^ ^43^ ^]^ Note that F50 delivered a lower *µ*_h_ value than F40, which could be attributed to its inferior film morphology. As seen from the surface morphology measured by atomic force microscopy (AFM) (Figures S9 and S10, Supporting Information), the F50 film displayed the roughest surface morphology with severe phase aggregation, which is not favorable for charge transport. Such a rough surface might result from its inferior solubility than the other polymers, as discussed earlier.

Figure 2b displays the n‐type transfer curves of these devices measured at a fixed *V*_DS_ of +100 V with the *V*_GS_ swept from −30 to +100 V. As seen, from F0 to F50, the transfer curve was similarly positively shifted but with a decreased current with an order of 10^2^. This change reveals the diminishing n‐type feature from F0 to F50, as also confirmed by their corresponding n‐type output curves (Figure S11, Supporting Information). The gradually decreased *µ*_e_ value from F0 to F50 can be ascribed to the upshifted LUMO level (Figure 1c), which increases the electron injection barrier with the Au electrode. The changes of electron mobility (*µ*_e_) values and the *V*_th_s of these devices are similarly portrayed in Figure 2c,d. Clearly, the various *µ*_h_/*µ*_e_ ratios of these polymers fine‐tune their ambipolarity, for which the *µ*_h_/*µ*_e_ ratio is increased remarkably from 1 for F0 to 25 for F50 (Figure 1a). We also measured the transistor performance of these devices under vacuum. The corresponding transfer curves (Figures S12 and S13, Supporting Information), output curves (Figures S14 and S15, Supporting Information), and device performance (Table S3, Supporting Information) are provided in the Supporting Information. Figure S16, Supporting Information, depicts the changes of *µ*_h_, *µ*_e_, and the *µ*_h_/*µ*_e_ ratio of these devices measured under vacuum. Their varying trends are comparable to those measured in an N_2_‐filled environment but with a more dominant n‐type feature, especially for F0 and F10. The above results clearly manifest the fine‐tuned ambipolarity between F0 and F50.

### 2.3 Volatile Memory Characteristics

Ambipolar transistors have been shown to possess memory characteristics due to the bipolar charge storing capability of the active channels.^[^ ^44^ ^]^ We subsequently investigated the memory characteristics of the F0–F50 devices, intending to understand the influence of polymer's ambipolarity. As shown in Figure S17, Supporting Information, obvious current–voltage hysteresis was observed for these devices measured under a dual‐sweep mode, clearly manifesting the bipolar charge trapping capability of these devices.^[^ ^34^, ^45^ ^]^ Owing to the balanced ambipolar charge transport properties, the F0 and F10 devices both exhibited a typical butterfly‐shape, large hysteresis. For F25–F50 with an unbalanced ambipolarity, the hysteresis was gradually reduced. We also measured the Kelvin probe force microscopy of the films of F0–F40. As shown in Figure S18, Supporting Information, F0 and F10 with higher ambipolarity exhibited a larger surface potential compared to F25 and F40 with lower ambipolarity. The decreasing ambipolarity from F0 to F40 results in decreased surface potential. Notably, the higher surface potential for polymers with higher ambipolarity verifies their larger hysteresis in the transfer curves.^[^ ^46^, ^47^ ^]^

**Figure 3:**
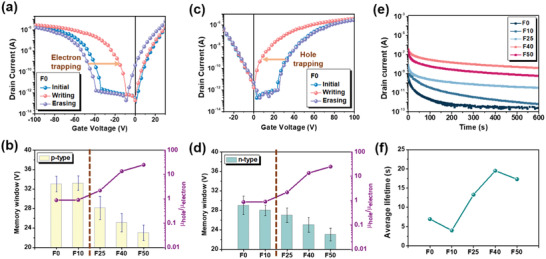
a) p‐Type memory characteristics of F0 device (writing: *V*_GS_ = 100 V for 1 s; erasing: *V*_GS_ = −100 V for 1 s) and b) the summary of the memory windows for the F0–F50 devices under a p‐type mode (*V*_DS_ = −100 V). c) n‐Type memory characteristics of F0 device (writing: *V*_GS_ = −100 V for 1 s; erasing: *V*_GS_ = +100 V for 1 s) and d) the summary of the memory windows for the F0–F50 devices under an n‐type mode (*V*_DS_ = +100 V). e) Charge retention time tests for the F0–F50 devices after biasing by a *V*_GS_ of +100 V for 3 s and f) the corresponding lifetimes calculated using a double exponential decay function as described in the content.

**Figure 4:**
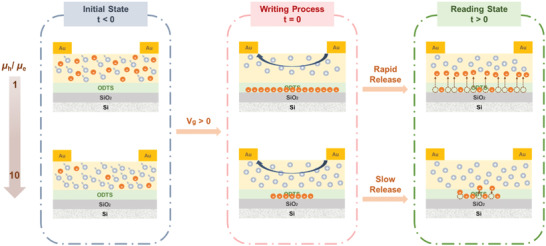
Illustration of charge trapping/releasing dynamic processes of the device under a p‐type mode and their correlation with the changes in ambipolarity.

We next examined the respective p‐type and n‐type memory characteristics of these transistors. Figure S19, Supporting Information, first presents the transfer curves of these devices under a p‐type mode (*V*_DS_ = −100 V). The transfer curves were positively shifted after applying a *V*_GS_ of 100 V for 1 s. This suggests that this programming induces electron trapping in the devices. However, such a written state can be erased and returned to the initial state by applying an opposite *V*_GS_ of −100 V (for 1 s). The shift of the *V*_th_s between these two states represents the so‐called memory window. As depicted in **Figure** **3**a, the F0 device delivered a large memory window of ≈33 V. Nevertheless, from F10 to F50, the device memory window gradually decreased and this trend is the opposite of the varying trend of polymer's ambipolarity (Figure 3b). In brief, a higher *µ*_h_/*µ*_e_ ratio (or a lower ambipolarity) results in a lower memory window, indicating that the weakened n‐type property for F25–F50 relative to F0–F10 induces less electron trapping. The mechanism of charge trapping in the channel is schematically depicted in **Figure** **4**. As illustrated in Figure 4 (left half), after programming the device with a large, positive *V*_GS_, electrons generated in the active channel diffuse to the octadecyltrimethoxysilane (ODTS) self‐assembled monolayer (SAM) and accumulate at the dielectric interface.^[^ ^35^ ^]^ The silanol groups of the ODTS SAM trap the accumulated electrons, acting like a temporary charge‐trapping floating gate.^[^ ^48^, ^49^ ^]^ Also, the long alkyl side chains of the ODTS SAM serve as tunneling barriers to stabilize the trapped electrons.^[^ ^50^ ^]^ The stronger electron transport properties provide more electrons injected into the active channel. The more electrons entering the channel increase the chance of being trapped by defects at the interface, leading to the occurrence of a larger *V*_th_ shift. In contrast, the reduced n‐type property of the active channel will induce lesser electrons trapped at the ODTS/dielectric interface, thereby lowering the shielding effect to the vertical electric field and a less change of *V*_th_.^[^ ^51^ ^]^ Moreover, the charge trapping density is estimated from the shift of the threshold voltage using the equation of Δ*n* = Δ*V*_th_ · *C*_i_/*e*, where *C*_i_ is the capacitance of the dielectric layer, Δ*V*_th_ is the *V*_th_ shift, and *e* is the elementary charge.^[^ ^33^ ^]^ As summarized in Table S4, Supporting Information, the polymers with higher ambipolarity show a higher charge trapping capability.

The memory performance of these transistors under an n‐type mode was also evaluated. The transfer curves measured under an n‐type mode (*V*_DS_ = +100 V) are shown in Figure S20, Supporting Information. The shift of *V*_th_ after programming (writing: *V*_GS_ = −100 V for 1 s; erasing: *V*_GS_ = 100 V for 1 s) was similarly observed. Similarly, from F0 to F50, the device memory window gradually decreased and its trend is also opposite to the trend of polymer's ambipolarity (Figure 3d). The reduced memory window mainly stems from the change in the written state. As shown, after writing, the transfer curve of the F0 device can shift to the negative‐voltage direction (Figure 3c). This change correlates with their gradually diminishing n‐type characteristics. As illustrated in Figure S21, Supporting Information, after programming the device with a large, negative *V*_GS_, holes generated in the active channel diffuse to the ODTS SAM and are trapped at the ODTS/dielectric interface.^[^ ^41^ ^]^ However, the gradual increasing p‐type characteristic from F0 to F50 adversely affects the electron transport in the active channel, leading to a decreased memory effect and memory window.

Given that holes are the major charge carriers from F0 to F50, we hereafter focus on studying the charge storage capabilities of these devices under the p‐type mode. Figure 3e shows the memory retention behaviors of these devices measured at a reading *V*_GS_ of −20 V after being biased by a *V*_GS_ of +100 V for ≈3 s. Benefitting from the highest *µ*_h_ value, the F40 device delivers not only the highest drain current among all of the devices but also the longest charge retention ability, revealing a superior charge storage capability than the other devices. Moreover, the F40 device exhibits the multilevel charge storage capability that is manipulated by giving different magnitudes of gate biases as shown in Figure S22a, Supporting Information. To clarify the charge storage capabilities of F0–F50, we calculate the charge retention lifetime of each device using the following decay function:^[^ ^52^ ^]^

(1) $$I_{\mathrm{DS}}=I_{\infty }\;+A_{1}e^{-t/t_{1}}+A_{2}e^{-t/t_{2}}$$

(2) $$t_{\mathrm{avg}}=\frac{\sum_{i=1}^{2}A_{i}t_{i}}{\sum_{i=1}^{2}A_{i}}$$

where *t* refers to the retention time after applying the gate bias, *t*_1_ and *t*_2_ are the respective lifetimes for the slow and rapid phases, *A*_1_ and *A*_2_ are the relative decay amplitudes, and *t*_avg_ refers to the average lifetimes. All the relevant parameters are summarized in Table S5, Supporting Information. The calculated average lifetimes (Figure 3f) reveal that the device's retention capability gradually increases from F0 to F40 and then shows a diminishing return. This reveals that the polymer owning a balanced *µ*_h_/*µ*_e_ ratio (i.e., high ambipolarity) exhibits a lower charge retention capability despite showing a larger memory window. Whereas, the polymer owning a lower ambipolarity, namely with an increased *µ*_h_/*µ*_e_ ratio, can possess a higher charge retention capability despite its smaller memory window.

The device memory retention capability is normally governed by the releasing speed of the trapped charges at the ODTS/dielectric interface to the active channel. In our case, the decay of charge retention time closely correlates with the polymer's *µ*_h_/*µ*_e_ ratio. For F0 and F10 owning high ambipolarity, the fast releasing of the trapped electrons, as evident from the fast decay of drain currents, results from their relatively low‐lying LUMO levels and high *µ*_e_ values (Figure 4 [right half]). The charge releasing speed gradually becomes slower from F10 to F40 as a result of the upshifting LUMO levels and the decreasing *µ*_e_ values (Figure 4 (right half)). As for the F50 device, the almost p‐type dominant polarity leads to the less trapped electrons and the consequently easier releasing of them, thereby yielding a reduced retention lifetime. The retention capabilities for the F0–F50 devices in the n‐type mode were also investigated and the trend was similarly governed by the *µ*_h_/*µ*_e_ ratio, as shown in Figure S22b, Supporting Information. The increasing *µ*_h_/*µ*_e_ ratio from F0 to F50 results in a faster releasing speed of the trapped holes, as is evident from the faster decay in the drain current. To the best of our knowledge, our results, for the first time, clearly demonstrate the influence of the energy levels and the *µ*_h_/*µ*_e_ ratio of ambipolar polymers on the memory retention characteristics for floating‐gate free transistors.

### 2.4 Distinctly Different Synaptic Behaviors

**Figure 5:**
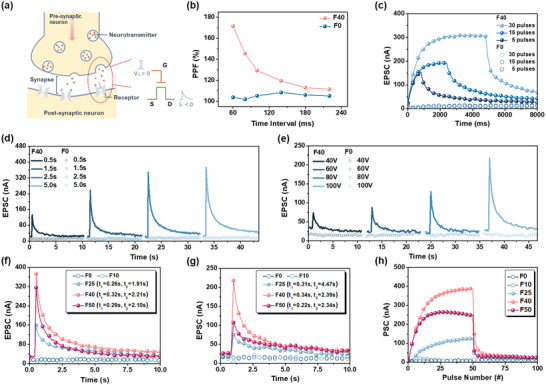
a) A schematic diagram of a biological synapse. b) The PPF index of the F0 and F40 devices as a function of presynaptic spike intervals (spike voltage: 80 V for 100 ms). c) EPSC of the F0 and F40 devices triggered by 5, 15, and 30 pulses (80 V for 100 ms per pulse and the pulse interval is 60 ms). d) EPSC of the F0 and F40 devices triggered with various gate pulse widths and f) the EPSC comparison of the F0–F50 devices measured at a fixed pulse width of 5 s. e) EPSC of the F0 and F40 devices triggered with various gate pulse biases and g) the EPSC comparisons of the F0–F50 measured at gate bias of 100 V. h) Current modulation of the F0–F50 devices under presynaptic stimuli for electrical potentiation (50 pulse number, presynaptic spike voltage: +80 V, pulse width: 100 ms, and pulse interval: 60 ms, respectively) and habituation (50 pulse number, presynaptic spike voltage: −80 V, pulse width: 100 ms, and pulse interval: 60 ms, respectively).

As introduced earlier, in a biological neutral network, a synapse is a connection between the axon of a presynaptic neuron and the dendrite of a post synaptic neuron. Synapses can convert a chemical signal into an electric impulse (**Figure** **5**a).^[^ ^5^ ^]^ In our case, the electron trapping process at the ODTS/dielectric interface can be analogous to the release of neurotransmitters in the synapses. Hence, the positive voltage spike and the resulting negative drain current correspond to the presynaptic input and the postsynaptic current (PSC), respectively. For a synaptic transistor, an EPSC triggered by two sequential potential pulses can be further increased in comparison with that applied by only a single potential pulse. This mimics the PPF behavior of a synapse and realizes the short‐term plasticity of neurons. Given the observed different memory retention characteristics between the F0 and F50 devices, we next explore their synaptic behaviors to clarify the influence of the polymer's ambipolarity on these emerging memory characteristics.

Since a longer memory retention characteristic could better accommodate a flexible spike modulation to mimic a synapse, we first examine the PPF behavior of the F40 device.^[^ ^29^, ^53^ ^]^ Herein, the EPSC was triggered by a pair of gate pulses (*V*_GS_ = +80 V for 100 ms and the pulse interval ranges from 60 to 220 ms) and recorded at a *V*_DS_ of −60 V. As displayed in Figure 5b, the F40 device delivers a conspicuous PPF behavior. It reaches a high PPF of 180% at the shortest pulse interval (60 ms) and shows an exponential decay as the pulse interval increases. Generally, the PPF behavior and the pulse interval have the following correlation:^[^ ^54^ ^]^

(3) $$\mathrm{PPF}\;=C_{1}\exp\left(-\frac{\Delta t}{\tau_{1}}\right)+C_{2}\exp\left(-\frac{\Delta t}{\tau_{2}}\right)$$

where Δ*t* is the pulse interval, *τ*_1_ and *τ*_2_ are the characteristic relaxation times for the rapid and slow phases, and *C*_1_ and *C*_2_ are the relative amplitudes. The rapid phase and slow phase can be referred to the release of trapped electrons from shallow and deep defects, respectively. As seen, the PPF behavior of the F40 device well fits with Equation (3), demonstrating its successful simulation of a synaptic transistor. In striking contrast, the F0 device did not show any PPF behavior (Figure 5b), just being a regular volatile transistor memory. Such a disparity between them suggests that the PPF performance closely correlates with device memory retention characteristics. In principle, when applying the first pulse to the device, electrons generated in the active channel will be trapped at the ODTS/dielectric interface and produce the EPSC. If the second pulse is soon applied after the first pulse, the trapped electrons basically do not have enough time to be completely released back to the channel layer; instead, more electrons will be trapped at the dielectric interface that enhances the EPSC and induces the PPF behavior, like the case of the F40 device. The negligible PPF behavior observed for the F0 device is apparently due to its short charge retention. The fast releasing of the trapped electrons after the first pulse counteracts the EPSC.

Given the conspicuous PPF response of the F40 device, we evaluate its energy consumption (*E*_spike_), which is an important index for neuromorphic computing, using the following equation:^[^ ^19^ ^]^

(4) $$E_{\mathrm{spike}}=V_{\mathrm{in}}\;\times \delta \times {\mathrm{PSC}}_{\mathrm{peak}}$$

where *V*_in_ and *δ* are the amplitude and the width of the input pulse, respectively, and PSC_peak_ refers to the value of the triggered EPSC. For our F40 device, *V*_in_ is 40 V, *δ* is 30 ms, and PSC_peak_ is 7.24 nA (Figure S23, Supporting Information), corresponding to an *E*_spike_ value of 8.7 nJ. This value is comparable to the values reported for electrically programmed synaptic devices.^[^ ^27^, ^28^, ^31^ ^]^ It should be noted that this value can be further reduced by using a dielectric layer with a higher capacitance. To probe this, we fabricated the F40 device using a 100 nm SiO_2_ dielectric layer, which has a larger capacitance (29 nF cm^−2^) than that (10 nF cm^−2^) of the 300 nm SiO_2_ dielectric layer. The resultant memory and synaptic characteristics of this device are presented in Figure S24, Supporting Information. As shown, the significant current–voltage hysteresis was similarly observed under a dual‐sweep mode (Figure S24a, Supporting Information). Importantly, as compared to the device using a 300 nm SiO_2_ dielectric layer, the memory retention behavior can be achieved by a drastically lower operating voltage of 30 V (Figure S24b, Supporting Information). Figure S24c–f, Supporting Information, shows its relevant synaptic behaviors, such as SVDP, spike‐duration‐dependent plasticity (SDDP), and potential/depression. Obviously, the operating voltage and speed are successfully reduced to be less than 30 V and 200 ms, respectively. Consequently, the energy consumption can be minimized to 2.7 nJ (*V*_in_ is 10 V, *δ* is 20 ms, and PSC_peak_ is 13.4 nA), showing a good potential for practical neuromorphic computing.

Since the synaptic memory mechanisms can be further classified into short‐term memory (STM) and long‐term memory (LTM) according to the retention time,^[^ ^53^ ^]^ we next examine the transition from STM to LTM of our device by adjusting the numbers of gate pulse (+80 V, time interval is 60 ms). Figure 5c compares the EPSC curves of the F0 and F40 devices. Again, the F0 device did not show any EPSC enhancement; whereas, the EPSC of the F40 device increased remarkably as increasing the pulse numbers. In addition, it can be observed that a longer time is needed to return to the initial state as increasing the pulse numbers, revealing a clear transition from STM to LTM. The EPSC curves of the other devices were also recorded and displayed in Figure S25, Supporting Information. As shown, from F0 to F50, a gradual increasing EPSC along with a more apparent transition from STM to LTM was clearly observed. This again confirms the critical role of the memory retention characteristics on the induced synaptic behaviors.

It has been known that unremitting presynaptic stimulations can enhance the depth of learning and memory level in the human brain, and this can be mimicked by SDDP. We thus recorded the SDDP curves of our devices by a spike voltage of +80 V and with various spike durations from 0.5 to 5 s, as shown in Figure S26, Supporting Information. Figure 5d compares the SDDP curves of the F0 and F40 devices. Clearly, in contrast to the no response of the F0 device, the F40 device delivered a remarkably enhanced EPSC after longer spike durations. It can reach a high current of 400 nA when the spike duration is 5 s. Obviously, increasing spike duration induces more electrons trapped at the dielectric interface and then triggers a higher EPSC. It is noteworthy that the increasing rate of EPSC with the spike duration gets slower at 2.5 s. It indicates that the charge trapping sites are saturated in the device. Figure 5f compares the difference in SDDP of the F0–F50 devices measured under a spike duration of 5 s. As can be seen, starting from the F25 device, the increase in EPSC became apparent and the F40 device possessed the most progressively enhanced EPSC. We calculated the decay lifetimes of the F25, F40, and F50 devices by fitting their SDDP curves with Equation (1) (Figure S27, Supporting Information). As depicted in Figure 5f, the *τ*_1_ values for the F25, F40, and F50 devices are 0.26, 0.32, and 0.29 s, and the *τ*_2_ values are 1.91, 2.21, and 2.10 s, respectively. Obviously, the F40 device exhibited the longest forgetting lifetime, benefitting from its longest memory retention capability (Figure 3e,f). This result again unveils that the increasing *µ*_h_/*µ*_e_ ratio of the polymer channel layer from 1 to 10 endows the derived device with more apparent synaptic behaviors. This slightly mismatched *µ*_h_/*µ*_e_ ratio prevents the fast releasing of the trapped electrons, thereby resulting in a certain degree of enhancement for the EPSC after the pulse stimulation.

Analogously, the SVDP of these devices was simulated, as presented in Figure S28, Supporting Information. As shown, even though the gate pulse voltages were increased, the F0 and F10 devices still showed no EPSC enhancement. However, starting from F25, the device began to exhibit an enhanced EPSC as the pulse voltages were increased, especially for the F40 device (Figure 5e). Figure 5g compares the difference in SVDP of the F0–F50 devices measured at a gate bias of 100 V and the decay lifetimes of the F25, F40, and F50 devices were similarly fitted using Equation (1) (Figure S29, Supporting Information). Notably, the data of the F25 device was not well fitted owing to its low EPSC. Except for this, the trend of lifetime calculated in the SVDP is similar to that in SDDP.

At last, we mimic the key characteristics of synaptic excitatory and inhibitory responses of our devices. Herein, the potentiation process consists of 50 consecutive positive pulses (*V*_GS_ = +80 V, pulse width = 100 ms, and pulse interval = 60 ms) while the depression process is made of 50 consecutive negative pulses (*V*_GS_ = −80 V, pulse width = 100 ms, and pulse interval = 60 ms). As shown in Figure 5h, the F0 and F10 devices still showed no clear response owing to their short memory retention features. However, apparent synaptic behaviors were observed starting from the F25 device and the F40 device delivered the most significant response. The reduced PSC of the F50 device can be attributed to its low electron mobility, leading to fewer electrons filling the trapping sites. Note that the PSC of these devices increased sharply after the first few pulses and then gradually reached saturation. This indicates that the charge trapping sites at the ODTS/dielectric interface are rapidly filled after the first few pulses and subsequently reach saturation after a few pulses. Moreover, both the excitatory and inhibitory processes are strongly dependent on the gate bias, spike width, and spike frequency. As shown in Figure S30, Supporting Information, as increasing the spike width and spike frequency, the EPSCs are clearly triggered to higher values. In the inhibitory process, when the values of negative gate biases are increased from −40 to −80 V and the pulse widths are raised from 60 to 100 ms, the inhibitory synaptic responses become more significant. These results affirm the close correlation between the memory retention characteristics of the transistor device and the synaptic behaviors. Because the memory retention characteristics are governed by the *µ*_h_/*µ*_e_ ratios and energy levels of the polymer channel layers in the floating‐gate free transistors, the critical role of the polymer's ambipolarity on the compromise between the volatile memories and artificial synapses can be concluded.

## 3 Conclusion

In summary, we describe a clear correlation among the *µ*_h_/*µ*_e_ ratio, the memory retention characteristic, and the synaptic behavior for the polymer channel layer in a floating‐gate free transistor by studying five DPP–benzotriazole dual‐acceptor random conjugated polymers (Figure 1d). We first show that the *µ*_h_/*µ*_e_ ratio gradually increases from F0 to F50 as increasing the content of the benzotriazole unit, leading to a gradual conversion from balanced ambipolarity (F0, F10) to a hole‐dominated carrier transport (F50). For the volatile transistor memory characteristics, the F0 device delivers the largest memory window but shows a poor charge retention behavior due to its balanced ambipolarity. As the polymer's *µ*_h_/*µ*_e_ ratio gradually increases (from F0 to F50), despite decreasing device memory window, the memory retention capability is gradually enhanced, especially for the F40 device with a *µ*_h_/*µ*_e_ ratio of 10. Surprisingly, the long transient retention capability makes the F40 device possess the most conspicuous artificial synaptic characteristics. In contrast, the F0 device with a poor charge retention capability does not show any synaptic response, while the F50 device with an almost p‐type dominant polarity affords decreased artificial synaptic characteristics compared to the F40 device. Our results demonstrate the influence of the energy levels and the *µ*_h_/*µ*_e_ ratios of ambipolar polymers on the volatile memories and artificial synapses for floating‐gate free transistors.

## 4 Experimental Section

### Materials

M1 was synthesized according to the procedures reported in the literature.^[^ ^46^ ^]^ M2 (4,7‐Bis(5‐bromo‐2‐thienyl)‐5,6‐difluoro‐2‐(2‐hexyldecyl)‐2H‐benzotriazole), and (3,3′‐difluoro‐[2,2′‐bithiophene]‐5,5′‐diyl)bis(trimethylstannane) were purchased from Luminescence Technology Corp and used without purification. 5‐Bromo‐2‐pyridinecarbonitrile, potassium *tert*‐butoxide, *tert*‐amyl alcohol, diisopropyl succinate, potassium carbonate, 18‐corwn‐6,9‐(bromomethyl)nonadecane, tris(o‐tolyl)phosphine, tris(dibenzylideneacetone)‐dipalladium, acetic acid, and the common solvents, including THF, dichloromethane, *N*,*N*‐dimethylformamide, chlorobenzene, methanol, hexane, and chloroform, for syntheses were purchased from Sigma‐Aldrich and used without purification. The synthetic details for the polymers (F0–F50) are described in the Supporting Information.

### Device Fabrication

A bottom‐gate/top‐contact transistor device was fabricated and used for testing the memory and synaptic characteristics in this study. The device was built on a highly n‐doped Si substrate with 300 nm thick SiO_2_ as the dielectric layer (*C*_i_ = 10 nF cm^−2^). All of the substrates were modified with a SAM of ODTS and sequentially rinsed and sonicated with toluene. The polymer solutions were prepared in chloroform with a concentration of 4 mg mL^−1^ and heated at 50 °C for ≈3 h. Afterward, the precursor solution was spin‐coated onto the substrate at 2000 rpm for 60 s, followed by annealing at 200 °C for 30–40 min inside a N_2_‐filled glove box. Finally, the contact gold electrodes with a thickness of 60 nm were thermally deposited under high vacuum (10^−6^ torr) through a shadow mask, with defined channel length (*L*) and width (*W*) of 50 and 1000 µm, respectively. According to the saturation region of a transistor device, the field‐effect mobility (*µ*) and the threshold voltage (*V*_th_) could be determined using the following equation:

(5) $$\;I_{\mathrm{DS}}=\frac{W}{2L}\;\mu C_{i}{\left(V_{\mathrm{GS}}-V_{\mathrm{th}}\right)}^{2}$$

where *I*_DS_ and *V*_GS_ were the source–drain current and the source‐gate voltage, respectively, and *C*_i_ was the capacitance of the 300 nm thick SiO_2_ dielectric that was measured per unit area.

### Characterization and Measurement

^1^H NMR spectra were characterized using Bruker DPX400 MHz and deuterated chloroform (CDCl_3_) was used as the solvent for monomer M1 and the target polymers. Microwave polymerization was conducted in a 5 mL vessel using a Biotage microwave reactor. The weight‐ and number‐average molecular weight (*M*_w_ and *M*_n_) values of the target polymers were measured using a SEC in Enshine SUPER CO‐150 with polystyrene gel columns (Styragel HR2 and styragel 4) eluted with THF at 1.0 mL min^−1^ calibrated by standard polystyrene. The TGA of the target polymers was measured using TA instruments Q50‐TG with a heating rate of 10 °C min^−1^ and their differential scanning calorimetry (DSC) was conducted using TA instruments DSC 25 with a same heating rate. To measure the properties of a polymer film, the polymer solution was spin‐coated onto a quartz substrate to form a uniform film and used for further investigations. The UV−vis absorption spectrum was obtained using a Hitachi U‐4100 spectrophotometer. CV was measured using a CHI 627E electrochemical analyzer with the three‐electrode method, wherein the indium tin oxide substrates coated with polymers, platinum wire, and Ag/AgCl, KCl (saturation) were, respectively, used as the working electrode, auxiliary electrode, and reference electrode, and the electrolyte was made of 0.1 m tetrabutylammonium perchlorate in freshly distilled acetonitrile. The surface morphology of the polymer film was analyzed using AFM. The electrical properties of the transistor memories were characterized using a Keithley 4200‐SCS semiconductor parameter analyzer (Tektronix) in a N_2_‐filled glove box at room temperature, and the synaptic characteristics were measured using Keithley 2634B.

## Conflict of Interest

The authors declare no conflict of interest.

## Supporting information

Supporting Information

## Data Availability

The data that support the findings of this study are available from the corresponding author upon reasonable request.
