# Supplementary material for: Tuning Ambipolarity of the Conjugated Polymer Channel Layers of Floating‐Gate Free Transistors: From Volatile Memories to Artificial Synapses
Source: Adv Sci (Weinh). 2022 Aug 19;9(31):2203025. doi: 10.1002/advs.202203025 (PMC9631064; doi:10.1002/advs.202203025)
Supplement: Supplementary file 1 — Supporting Information [file ADVS-9-2203025-s001.pdf]

ADVANCED SCIENCE
Open Access

Supporting Information

for *Adv. Sci.*, DOI 10.1002/advs.202203025

Tuning Ambipolarity of the Conjugated Polymer Channel Layers of Floating-Gate Free Transistors: From Volatile Memories to Artificial Synapses

Yu-Ting Yang, Ying-Sheng Wu, Waner He, Hsin-Chiao Tien, Wei-Chen Yang, Tsuyoshi Michinobu, Wen-Chang Chen, Wen-Ya Lee and Chu-Chen Chueh**

Supporting Information

Tuning Ambipolarity of the Conjugated Polymer Channel Layers of Floating-Gate Free Transistors: From Volatile Memories to Artificial Synapses

Yu-Ting Yang,[a] Ying-Sheng Wu,[a] Waner He,[b] Hsin-Chiao Tien,[d] Wei-Chen Yang,[a,c] Tsuyoshi Michinobu,[b] Wen-Chang Chen,[a,c] Wen-Ya Lee,[d] and Chu-Chen Chueh[a,c*]*

[a] Y.-T. Yang, Y.-S. Wu, W.-C. Yang, Prof. W.-C. Chen and Prof. C.-C. Chueh
Department of Chemical Engineering, National Taiwan University, Taipei 10617, Taiwan

[b] W. He and Prof. T. Michinobu
Department of Materials Science and Engineering, Tokyo Institute of Technology, 2-12-1 Ookayama, Meguro-ku, Tokyo 152-8552, Japan

[c] W.-C. Yang, Prof. W.-C. Chen and Prof. C.-C. Chueh
Advanced Research Center of Green Materials Science and Technology, National Taiwan University, Taipei, 10617, Taiwan.

[d] H.-C. Tien and Prof. W.-Y. Lee
Research and Development Center for Smart Textile Technology and Department of Chemical Engineering and Biotechnology, National Taipei University of Technology, Taipei 106, Taiwan

*Corresponding author. E-mail: cchueh@ntu.edu.tw; wenyalee@mail.ntut.edu.tw

Keywords: Transistor memory; floating-gate free; conjugated polymers; ambipolarity; synaptic transistor

General Procedure for the Polymerization

1/0.9/0.75/0.6/0.5 molar ratios of M1 with 0/0.1/0.25/0.4/0.5 molar ratios of M2 were used to synthesize F0/F10/F25/F40/F50. Both of M1 and M2 together with $Pd_2(dba)_3$ and P(*o*-tolyl)$_3$ (both 0.03 equiv. with respect to the monomer) were dissolved in chlorobenzene and sealed in a microwave vessel in a N_2-filled glovebox. The reaction temperature for the polymerization was set at 140 ^oC in the beginning and was gradually risen to 160 ^oC with an increasing rate of 10 ^oC/min. The polymerization time was 90 min. The end-capping process

was proceeded at 160 °C for 15 min by sequentially adding 2-(tributylstannyl)thiophene and 2-bromothiophene (both 1.1 equiv. with respect to the monomer). After cooling to room temperature, the mixture was poured into methanol and the precipitate was collected. Soxhlet extraction was then conducted sequentially with methanol, acetone, and hexane. Finally, the product was extracted by chloroform and was recovered as solid by precipitation from methanol. The polymers were obtained by filtration and dried under vacuum.

F0. Anal. Calcd. for [$C_{64}H_{90}F_2N_4O_2S_2$]: C, 73.1; H, 8.8; N, 5.3; S, 6.1. Found: C, 68.7, H, 8.7; N, 4.5; S, 6.1. Molecular weight evaluated by SEC with tetrahydrofuran: M_n = 37.9 kDa, M_w = 91.9 kDa, $Đ$ = 2.42

F10. Anal. Calcd. for [$C_{61.4}H_{84.9}F_{2.2}N_{3.9}O_{1.8}S_{2.2}$]: C, 72.4; H, 8.4; N, 5.4; S, 6.9. Found: C, 69.6, H, 8.2; N, 4.8; S, 8.3. Molecular weight evaluated by SEC with tetrahydrofuran: M_n = 25.1 kDa, M_w = 58.3 kDa, $Đ$ = 2.32

F25. Anal. Calcd. for [$C_{57.5}H_{77.25}F_{2.5}N_{3.75}O_{1.5}S_{2.5}$]: C, 71.0; H, 7.9; N, 5.4; S, 8.2. Found: C, 67.8, H, 7.6; N, 4.8; S, 10.0. Molecular weight evaluated by SEC with tetrahydrofuran: M_n = 35.1 kDa, M_w = 67.5 kDa, $Đ$ = 1.92

F40. Anal. Calcd. for [$C_{53.6}H_{69.6}F_{2.8}N_{3.6}O_{1.2}S_{2.8}$]: C, 69.5; H, 7.5; N, 5.5; S, 9.7. Found: C, 67.2, H, 7.3; N,4.9; S, 11.0. Molecular weight evaluated by SEC with tetrahydrofuran: M_n = 24.2 kDa, M_w = 57.6 kDa, $Đ$ = 2.38

F50 Anal. Calcd. for [$C_{51}H_{64.5}F_3N_{3.5}O_1S_3$]: C, 68.4; H, 7.2; N, 5.5; S, 10.7. Found: C, 66.9, H, 7.0; N,4.9; S, 11.9. Molecular weight evaluated by SEC with tetrahydrofuran: M_n = 27.7 kDa, M_w = 81.0 kDa, $Đ$ = 2.93

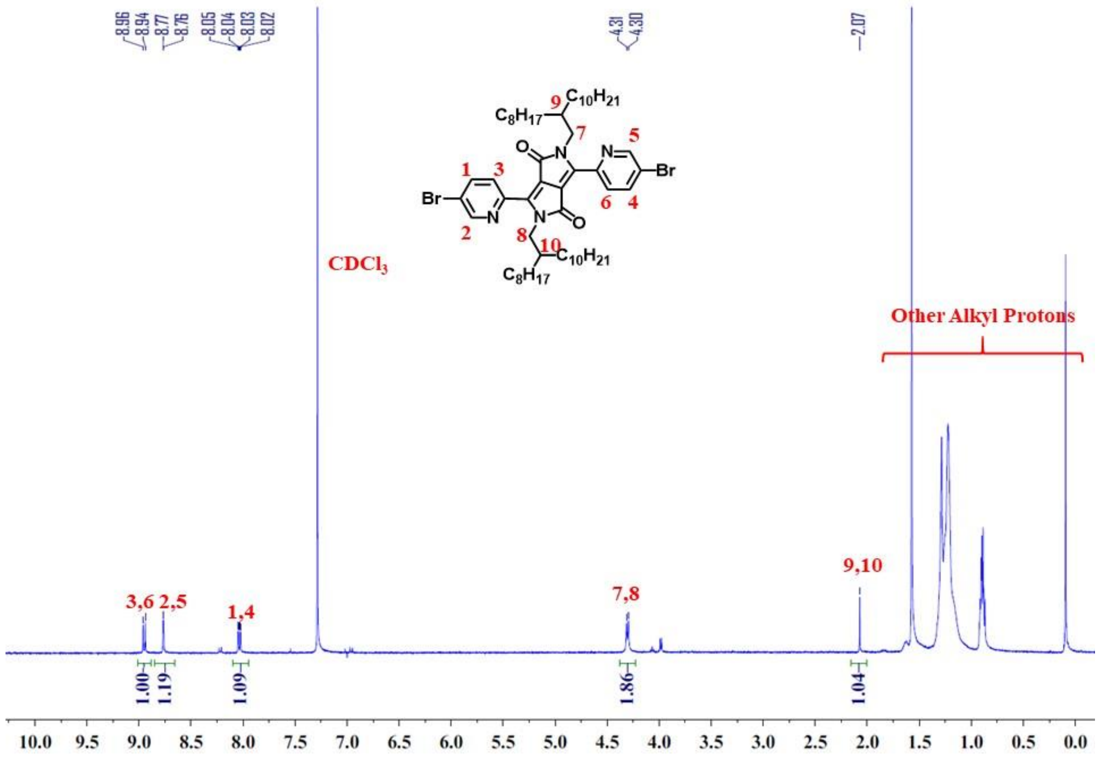

Figure S1.^{1}H-NMR spectrum of M1 in $CDCl_3$.

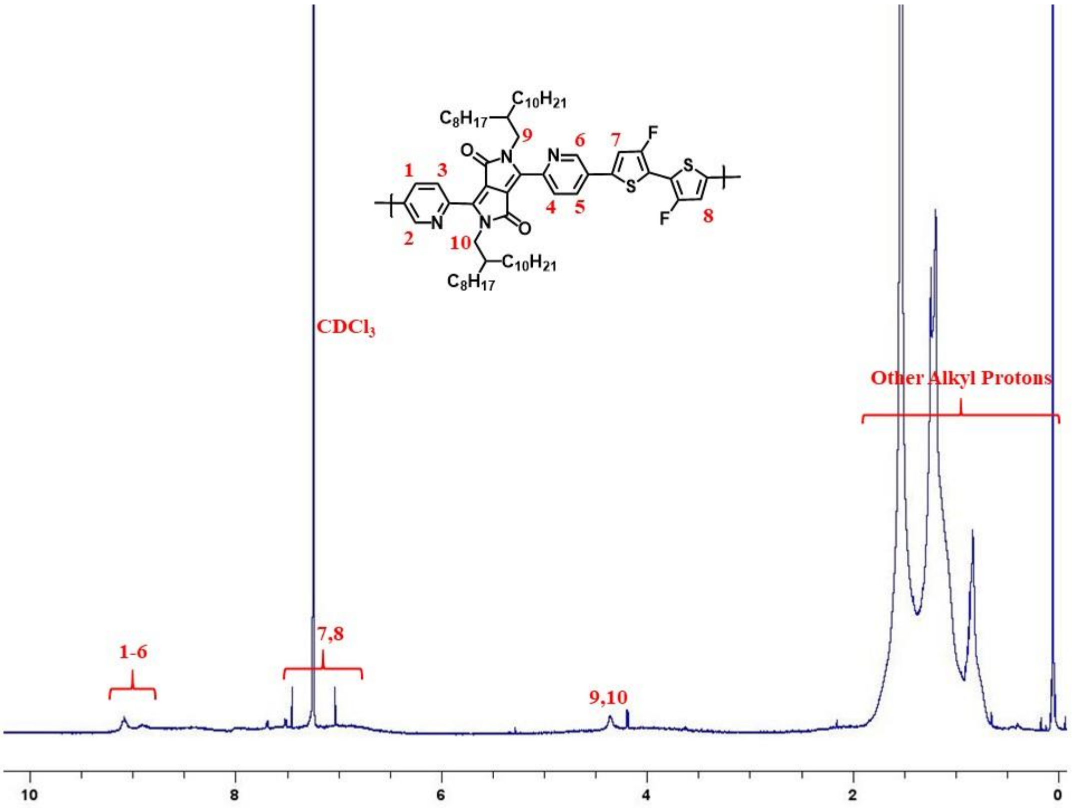

Figure S2. ^{1}H-NMR spectrum of F0 in $CDCl_3$.

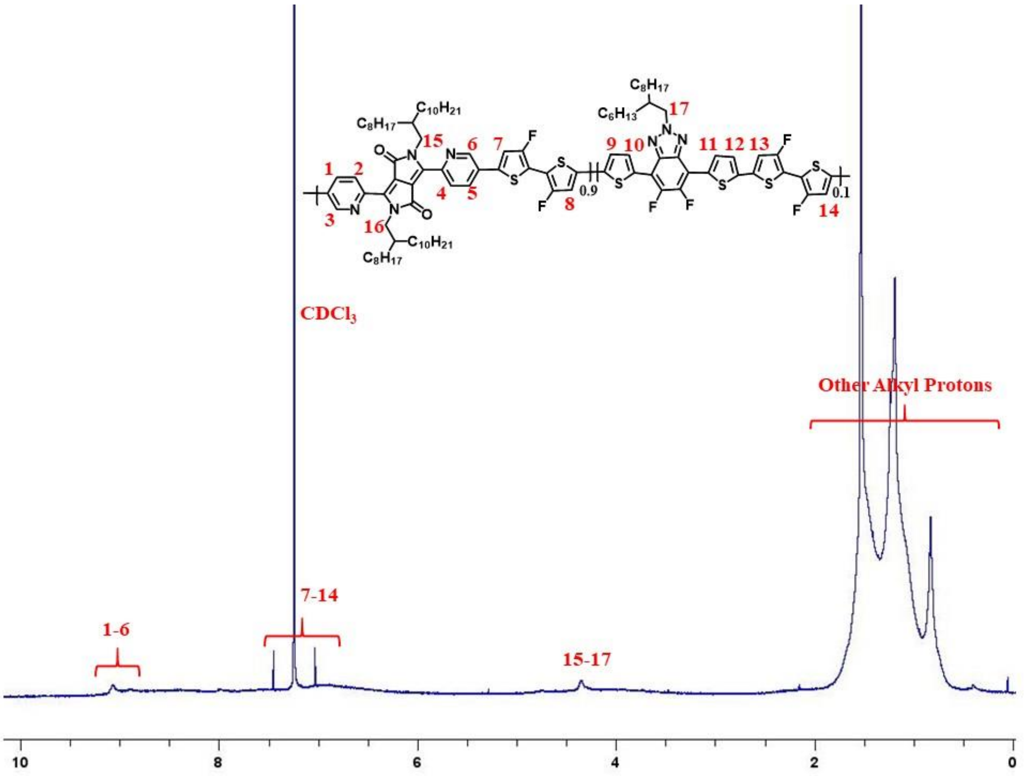

Figure S3. ^{1}H-NMR spectrum of F10 in $CDCl_3$.

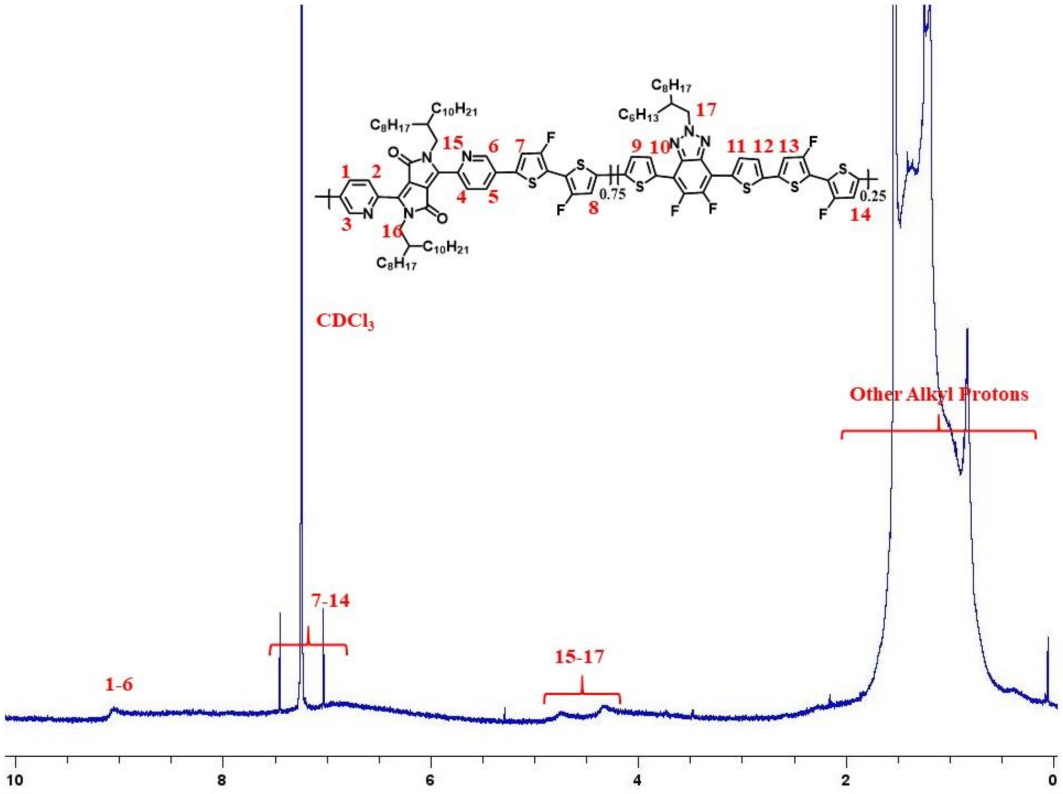

Figure S4. ^{1}H-NMR spectrum of F25 in $CDCl_3$.

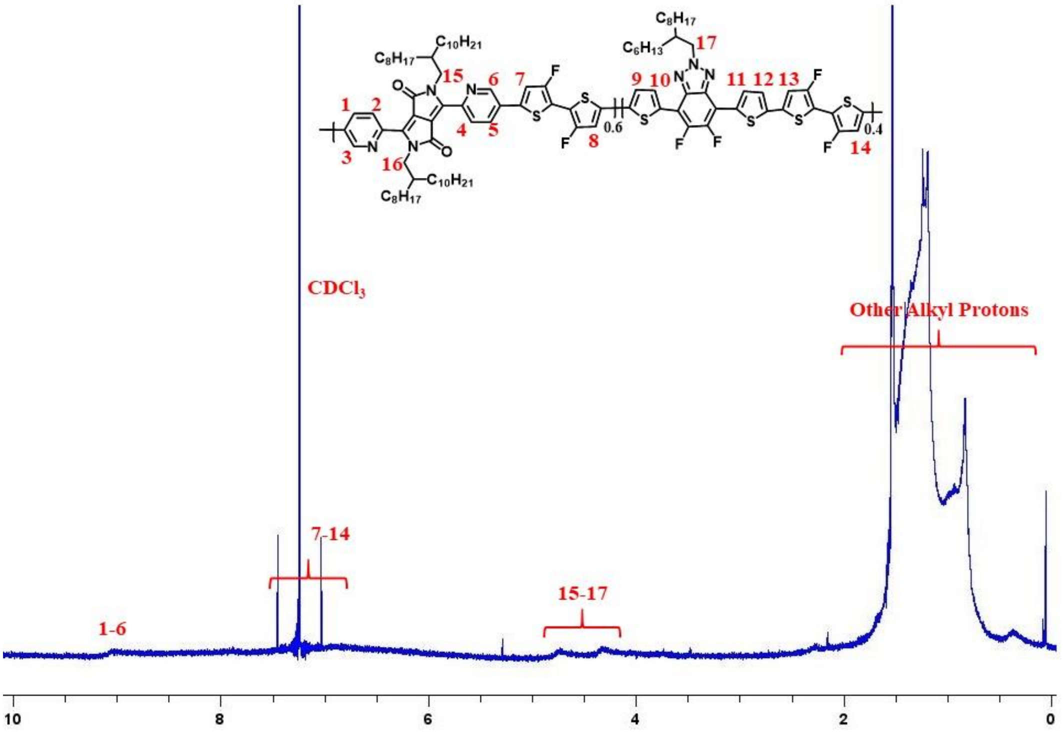

Figure S5. ^{1}H-NMR spectrum of F40 in $CDCl_3$.

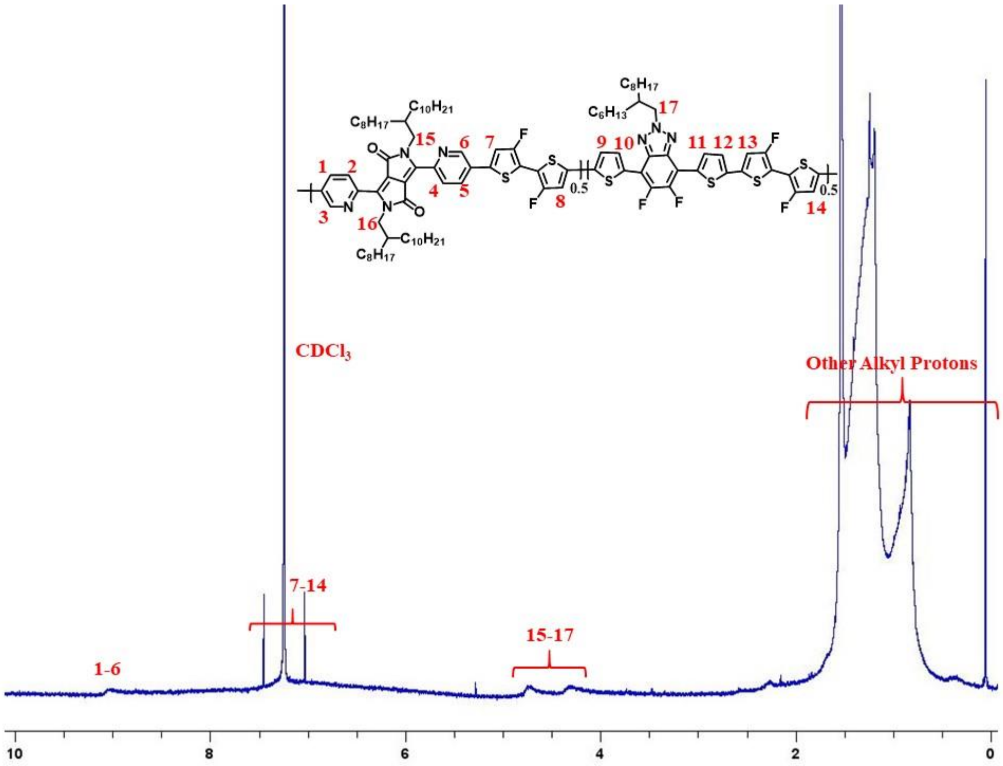

Figure S6. ^{1}H-NMR spectrum of F50 in $CDCl_3$.

Table S1. Physical, optical, and electrical properties of F0-F50.

	M_n (kDa)^a	M_w (kDa)^a	$Đ^a$	$T_{d,5\%}$ (°C)	HOMO (eV)^b	LUMO (eV)^b	E_g (eV)
**F0**	37.9	91.9	2.42	320	5.69	4.01	1.68
**F10**	25.1	58.3	2.32	360	5.59	3.95	1.64
**F25**	35.1	67.5	1.92	370	5.48	3.80	1.68
**F40**	24.2	57.6	2.38	360	5.42	3.78	1.64
**F50**	27.7	81.0	2.93	370	5.38	3.70	1.68

^{a} M_n, M_w, and $Đ$ were measured by a THF-eluted SEC. ^{b} CV determined using Fc/Fc^+ as an internal potential reference. ^{c} Estimated by LUMO = HOMO + E_g, where HOMO = -4.4 – $E_{ox.onset}$ calculated by CV and E_g = 1240 / λ_{onset} acquired by UV absorption spectra.

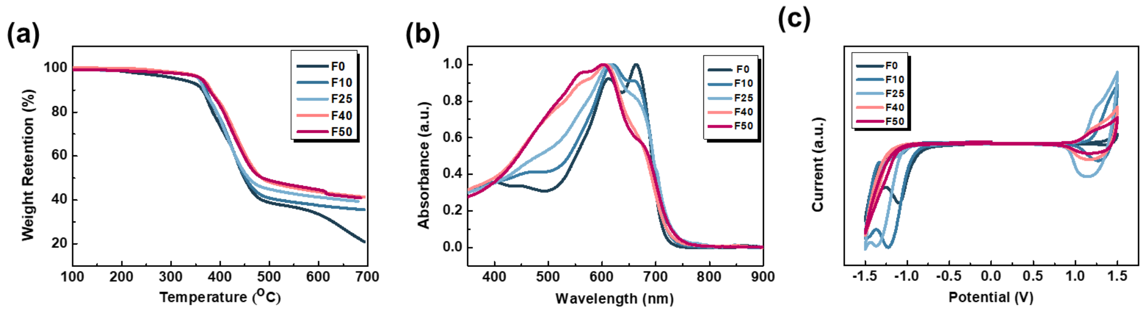

Figure S7. (a) TGA curves, (b) solution UV-Vis absorption spectra (in chlorobenzene), and (c) film CV characteristics of F0-F50.

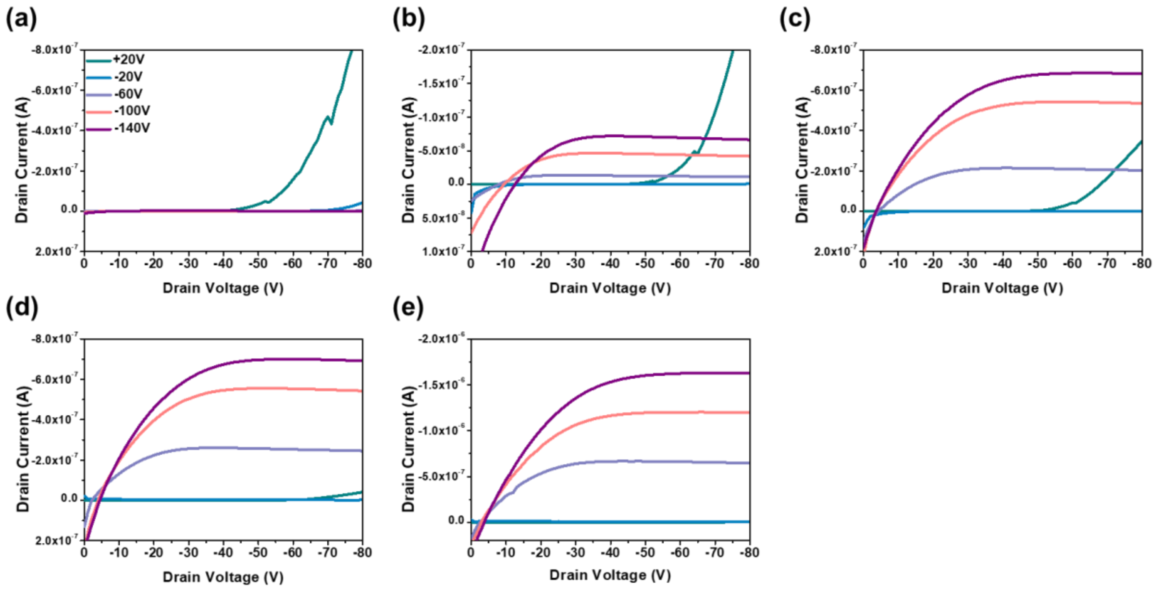

Figure S8. p-Type output curves measured in a N_2-filled glove box for the (a) F0, (b) F10, (c) F25, (d) F40, and (e) F50 transistor devices.

Table S2. FET performance of F0-F50 transistor devices measured in a N_2-filled glove box.[a]

	$\mu_{e,avg}$ ($cm^2V^{-1}s^{-1}$)	I_{on}/I_{off}	$V_{th,avg}$ (V)	$\mu_{h,avg}$ ($cm^2V^{-1}s^{-1}$)	I_{on}/I_{off}	$V_{th,avg}$ (V)
**F0**	2.4×10^{-2} ($\pm3.2\times10^{-3}$)	10^5-10^6	51.7±2.9	2.1×10^{-2} ($\pm4.7\times10^{-3}$)	10^6-10^7	-52.5±2.2
**F10**	2.2×10^{-2} ($\pm7.8\times10^{-3}$)	10^5-10^6	48.0±2.6	1.9×10^{-2} ($\pm6.7\times10^{-3}$)	10^6-10^7	-43.9±2.4
**F25**	1.5×10^{-2} ($\pm5.7\times10^{-3}$)	10^5-10^6	50.0±1.6	3.3×10^{-2} ($\pm8.9\times10^{-3}$)	10^5-10^6	-33.8±2.7
**F40**	2.5×10^{-3} ($\pm9.1\times10^{-4}$)	10^5-10^6	59.0±1.9	3.4×10^{-2} ($\pm7.6\times10^{-3}$)	10^5-10^6	-23.8±1.5
**F50**	1.0×10^{-3} ($\pm3.5\times10^{-4}$)	10^5-10^6	60.1±2.7	2.5×10^{-2} ($\pm4.6\times10^{-3}$)	10^5-10^6	-13.2±2.1

[a] FET performance was averaged from at least six devices

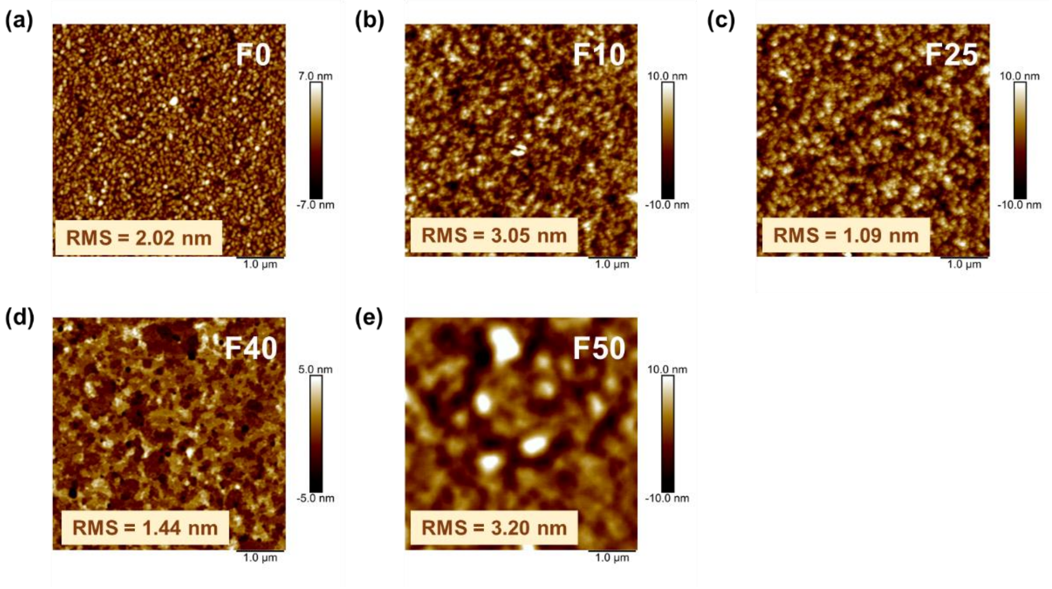

Figure S9. The AFM height images of the films of (a) F0, (b) F10, (c) F25, (d) F40, and (e) F50 with the corresponding root-mean-square (RMS) roughness value.

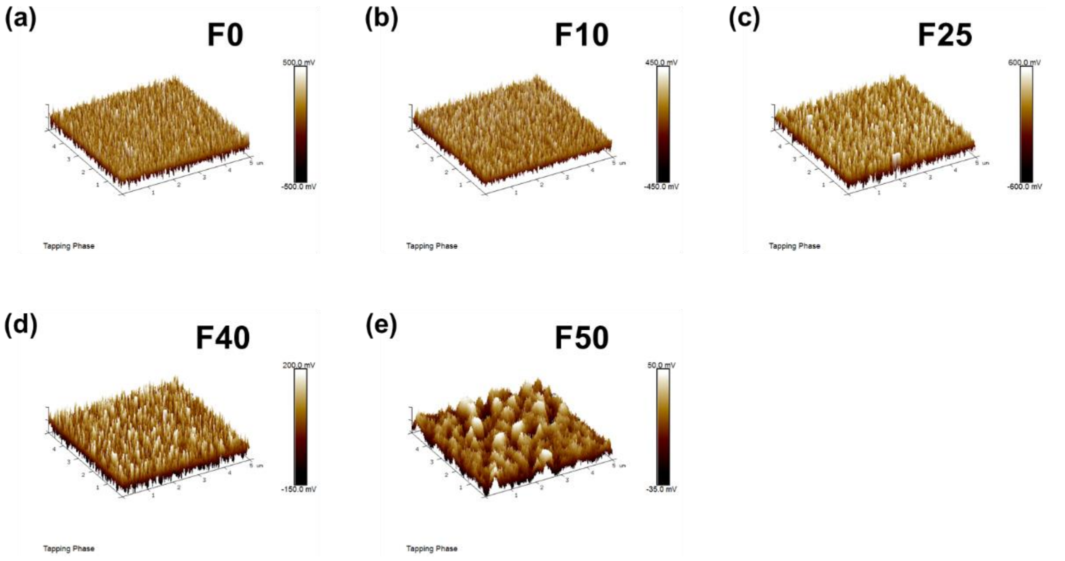

Figure S10. The AFM 3D phase images of the films of (a) F0, (b) F10, (c) F25, (d) F40, and (e) F50.

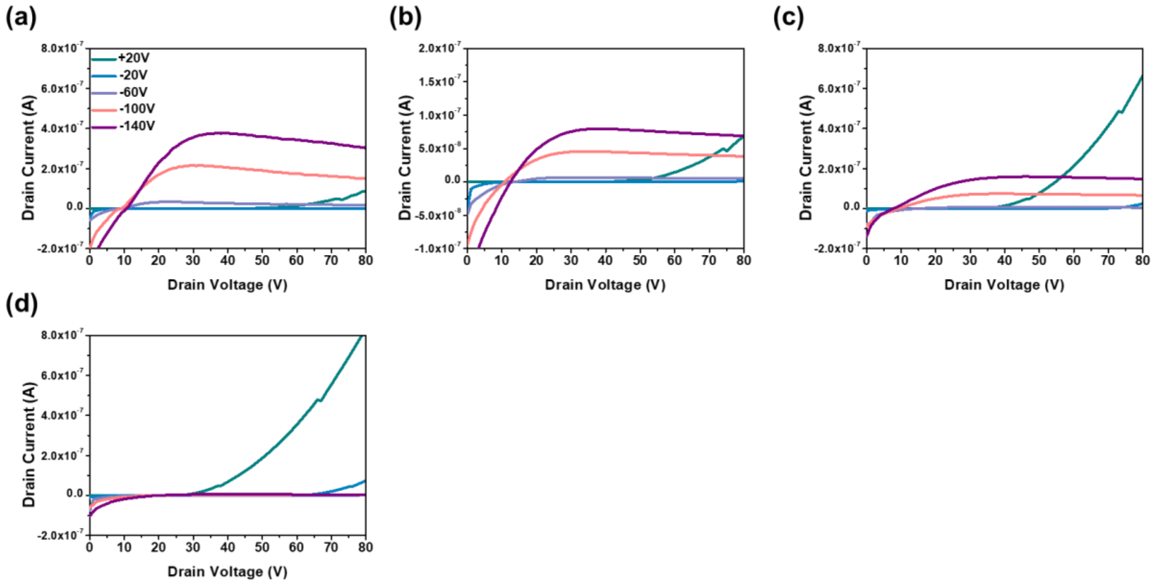

Figure S11. n-Type output curves measured in a N_2-filled glove box for the (a) F0, (b) F10, (c) F25, and (d) F40 transistor devices.

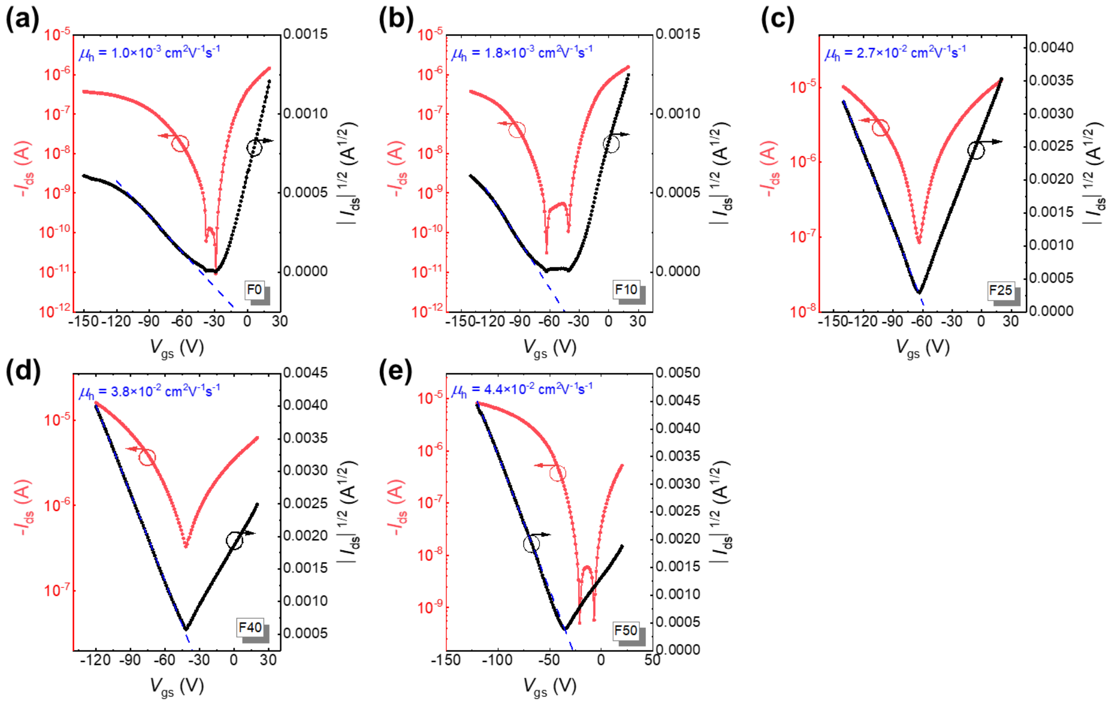

Figure S12. p-Type transfer curves of (a) F0, (b) F10, (c) F25, (d) F40, and (e) F50 transistor devices measured under vacuum.

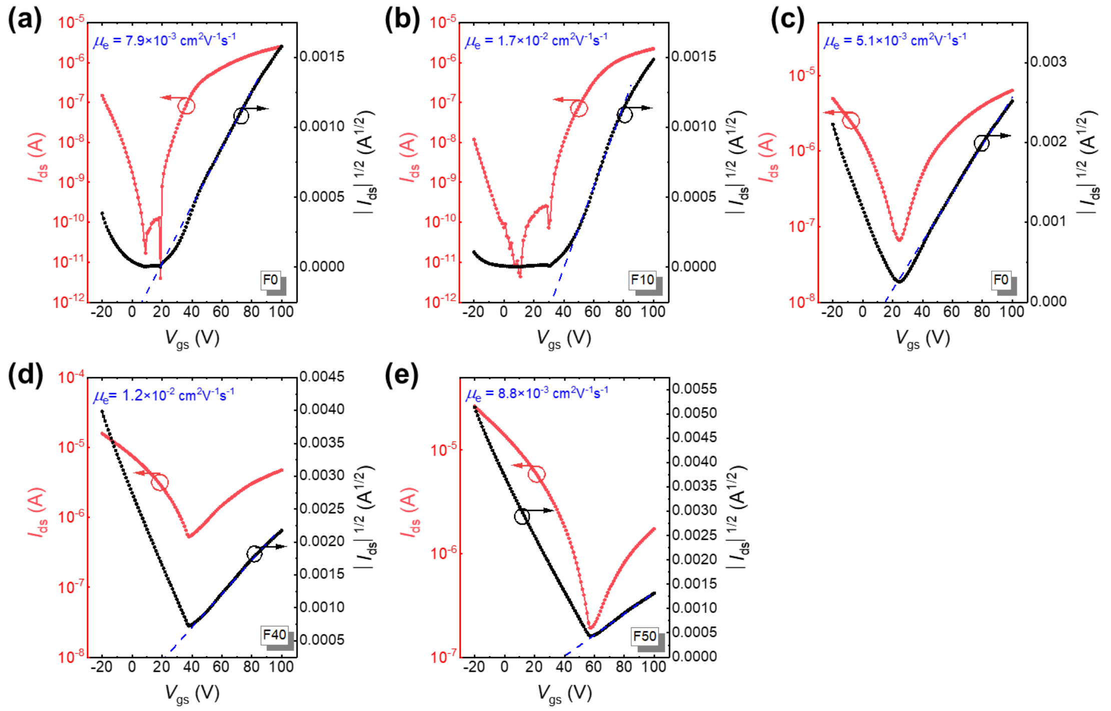

Figure S13. n-Type transfer curves of (a) F0, (b) F10, (c) F25, (d) F40, and (e) F50 transistor devices measured under vacuum.

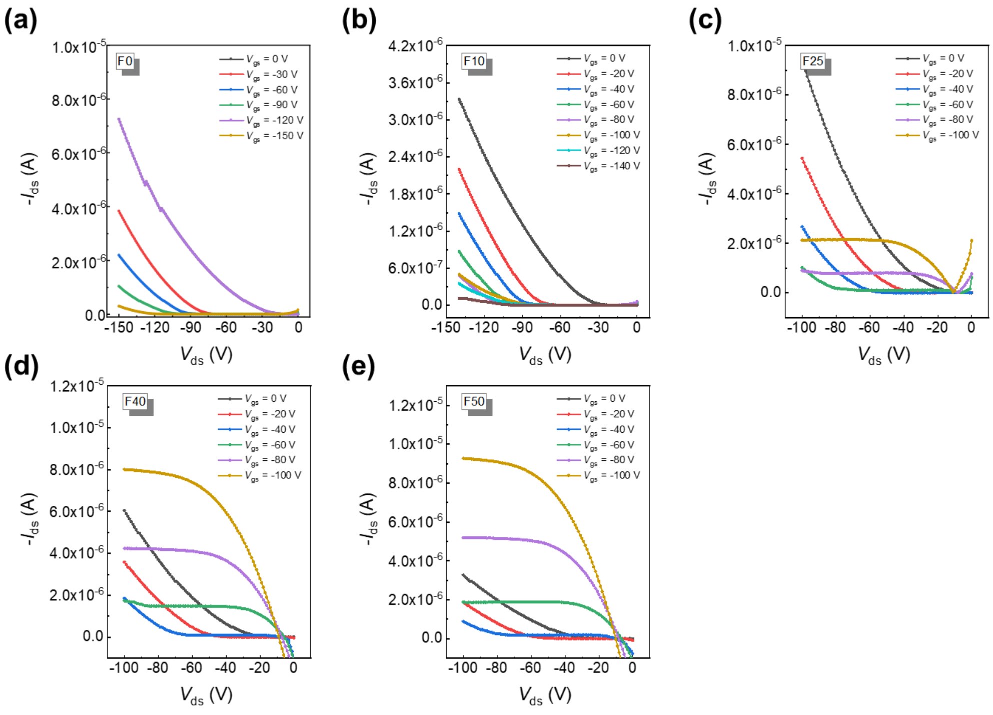

Figure S14. p-Type output curves measured under vacuum for the (a) F0, (b) F10, (c) F25, (d) F40, and (e) F50 transistor devices.

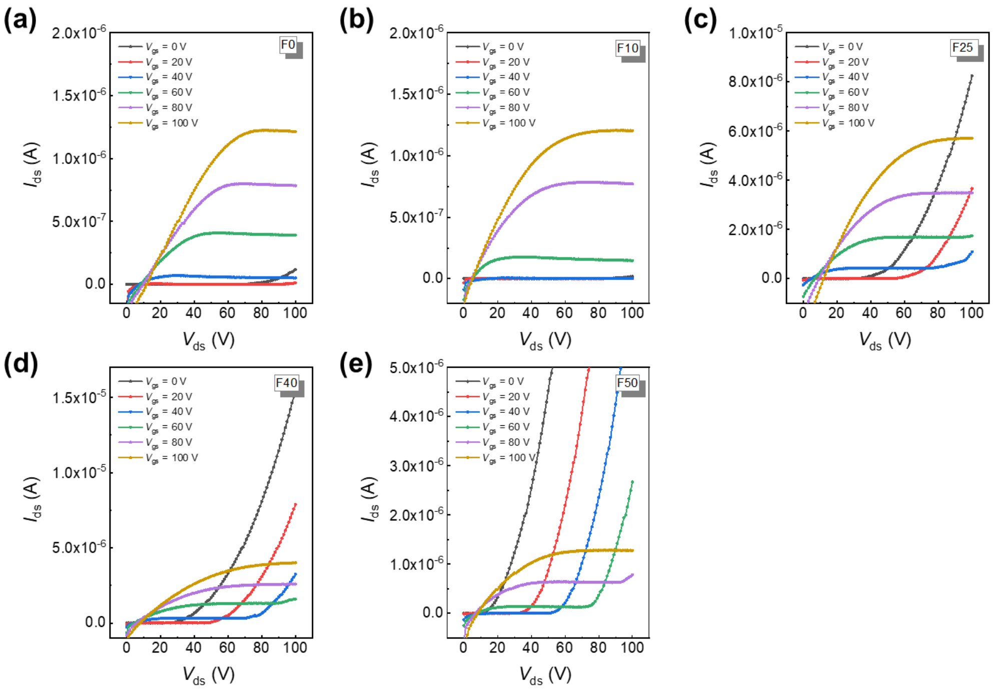

Figure S15. n-Type output curves measured under vacuum for the (a) F0, (b) F10, (c) F25, (d) F40, and (e) F50 transistor devices.

Table S3. FET performance of F0-F50 transistor devices under vacuum chamber at a pressure of ~10^{-5} mbar.[a]

	$\mu_{e,avg}$ ($cm^2V^{-1}s^{-1}$)	I_{on}/I_{off}	$V_{th,avg}$ (V)	$\mu_{h,avg}$ ($cm^2V^{-1}s^{-1}$)	I_{on}/I_{off}	$V_{th,avg}$ (V)
**F0**	6.4×10^{-3} ($\pm1.1\times10^{-3}$)	10^4-10^5	15.6±5.6	6.0×10^{-4} ($\pm3.5\times10^{-4}$)	10^4-10^5	-37.9±9.3
**F10**	6.9×10^{-3} ($\pm4.8\times10^{-3}$)	10^4-10^5	30.1±7.8	8.0×10^{-4} ($\pm7.9\times10^{-4}$)	10^4-10^5	-71.2±23.2
**F25**	1.1×10^{-2} ($\pm5.6\times10^{-3}$)	10^1-10^2	23.4±6.7	1.8×10^{-2} ($\pm6.9\times10^{-3}$)	10^2-10^3	-56.4±16.7
**F40**	1.0×10^{-2} ($\pm2.3\times10^{-3}$)	10^1-10^2	23.5±7.7	3.2×10^{-2} ($\pm4.7\times10^{-3}$)	10^1-10^2	-30.9±7.5
**F50**	4.8×10^{-3} ($\pm3.8\times10^{-3}$)	10^1-10^2	39.1±6.9	3.9×10^{-2} ($\pm4.2\times10^{-3}$)	10^3-10^4	-27.4±7.9

[a] FET performance was averaged from at least six devices.

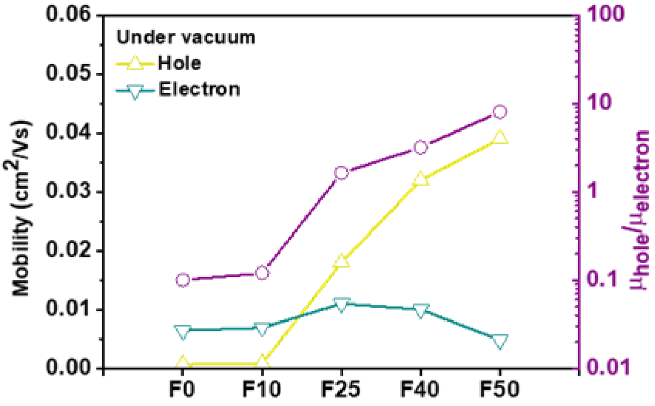

Figure S16. Summary of μ_h, μ_e, and the μ_h/μ_e ratio of F0-F50 transistor devices measured under vacuum.

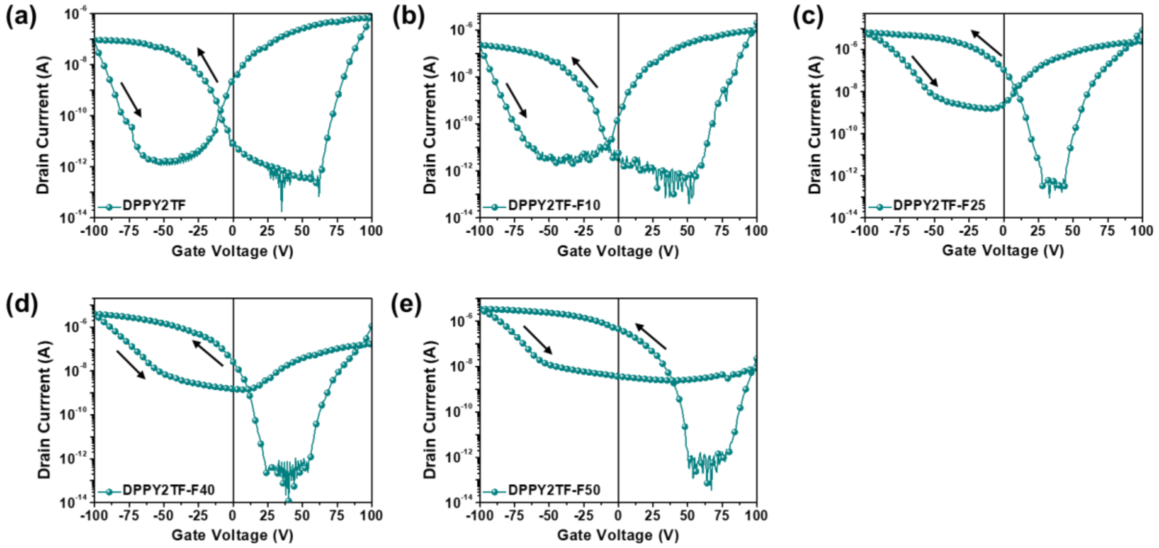

Figure S17. Current-voltage hysteresis for the (a) F0, (b) F10, (c) F25, (d) F40, and (e) F50 transistor devices measured under a dual-sweep model.

.

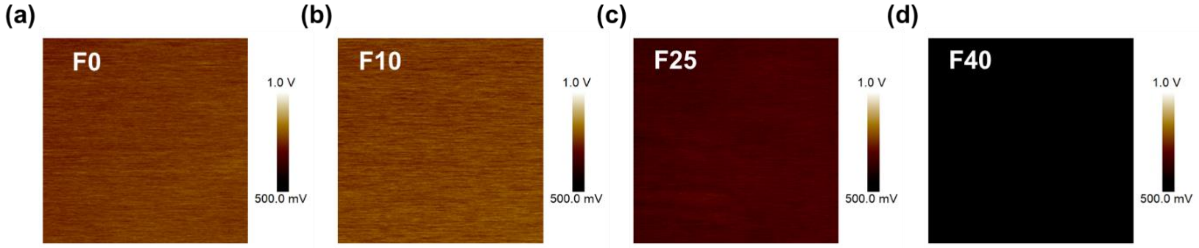

Figure S18. The KPFM images of the films of (a) F0, (b) F10, (c) F25, and (d) F40.

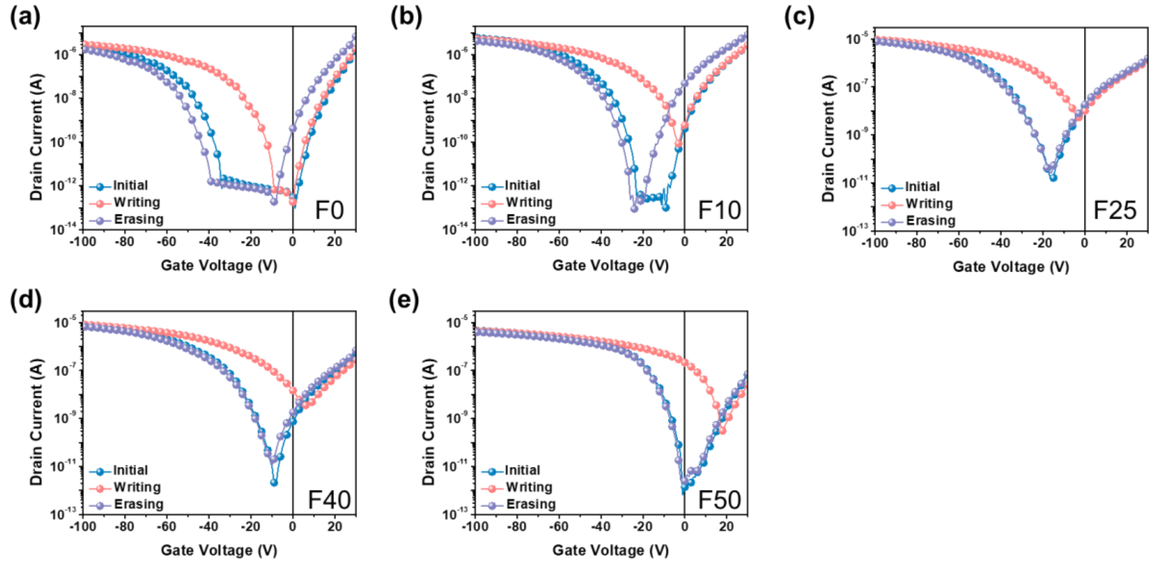

Figure S19. p-Type memory characteristics for the (a) F0, (b) F10, (c) F25, (d) F40, and (e) F50 transistor devices. (Writing: V_{GS} = 100 V for 1 s; erasing: V_{GS} = -100 V for 1s).

Table S4. Memory performance of the F0-F50 transistor devices.[a]

	$\Delta V_{th,\ p\text{-}type}$ (V)	$\Delta n_{p\text{-}type}$ (cm^{-2})	$\Delta V_{th,\ n\text{-}type}$ (V)	$\Delta n_{n\text{-}type}$ (cm^{-2})
**F0**	33.5±1.6	2.09×10^{12}	29.7±1.9	1.85×10^{12}
**F10**	33.2±1.5	2.08×10^{12}	28.7±1.1	1.79×10^{12}
**F25**	28.1±2.4	1.76×10^{12}	27.2±1.5	1.70×10^{12}
**F40**	25.1±1.9	1.57×10^{12}	25.7±1.5	1.61×10^{12}
**F50**	23.9±1.6	1.49×10^{12}	23.8±1.3	1.49×10^{12}

[a] FET performance was averaged from at least six devices.

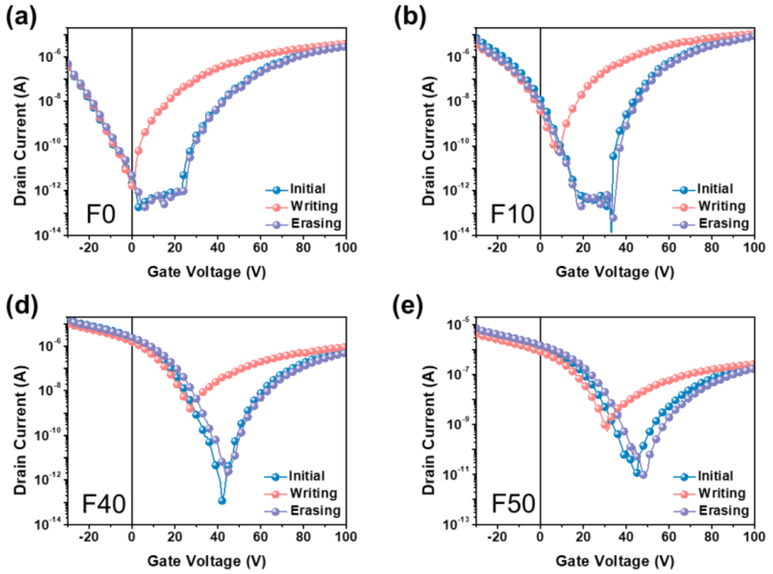

Figure S20. n-Type memory characteristics for the (a) F0, (b) F10, (c) F25, (d) F40, and (e) F50 transistor devices. (writing: V_{GS} = -100V for 1s; erasing: V_{GS} = 100V for 1s).

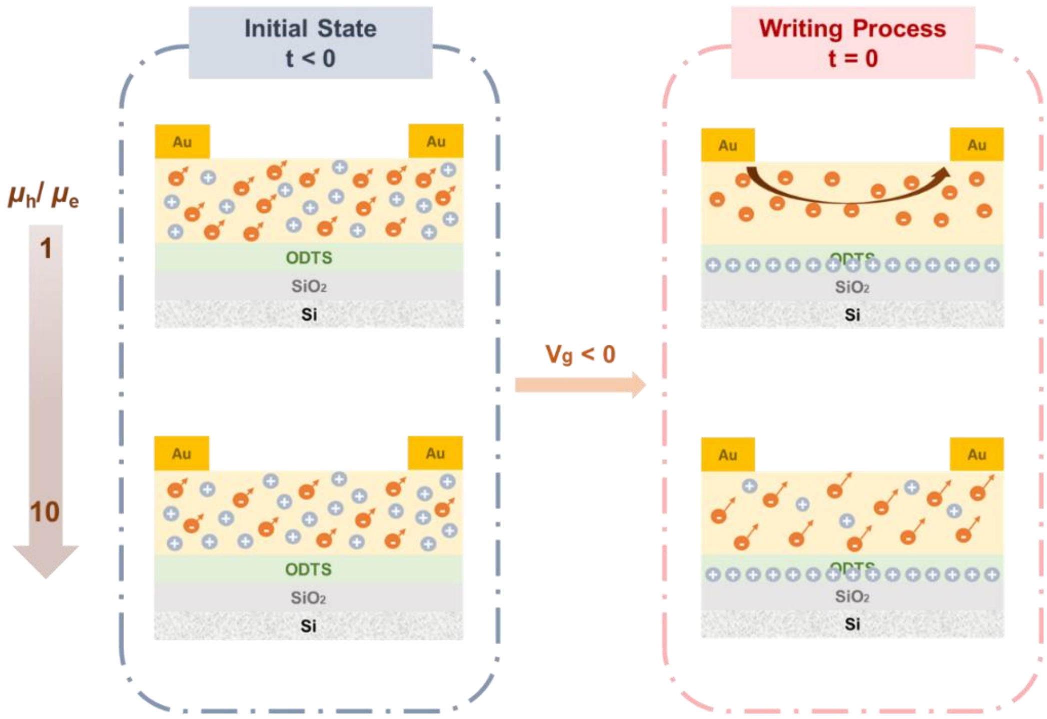

Figure S21. Illustration of the charge-trapping mechanism of the F0-F50 devices under an n-type mode (V_{DS} = +100 V) with the change of polymer's ambipolarity.

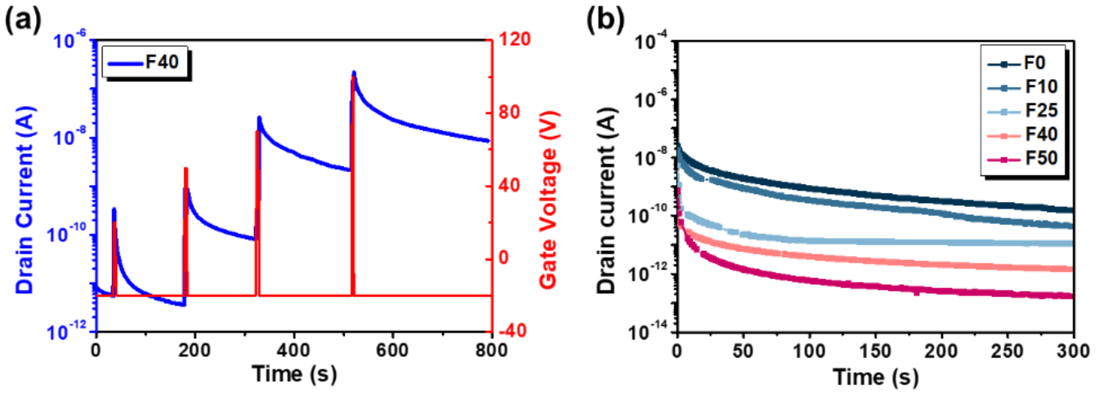

Figure S22. (a) Multilevel charge retention behaviors of the F40 device (writing: V_{GS} = 20, 50, 70, and 100 V for 3 s). (b) Charge retention time tests in the n-type mode (V_{DS} = 80V) for the F0-F50 devices after biasing by a V_{GS} of -100 V for 3 s.

Table S5. Retention characteristics under the p-type mode of the F0-F50 transistor devices.

	A_1 (A)	t_1 (s)	A_1 (A)	t_1 (s)	t_{avg} (s)
**F0**	1.15×10^{-10}	17.1	3.34×10^{-10}	3.45	6.95
**F10**	6.06×10^{-10}	12.0	1.77×10^{-9}	1.29	4.01
**F25**	1.64×10^{-8}	36.7	3.84×10^{-8}	3.27	13.3
**F40**	1.06×10^{-7}	43.9	1.72×10^{-7}	4.53	19.5
**F50**	2.55×10^{-7}	21.3	6.65×10^{-8}	1.80	17.3

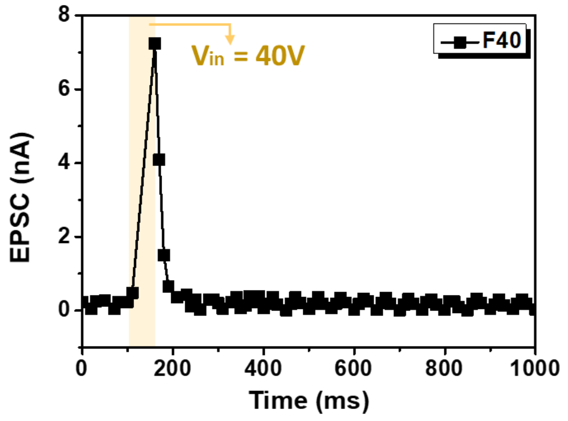

Figure S23. EPSC of the F40 device triggered by a single pulse with amplitude of +40 V and width of 30 ms.

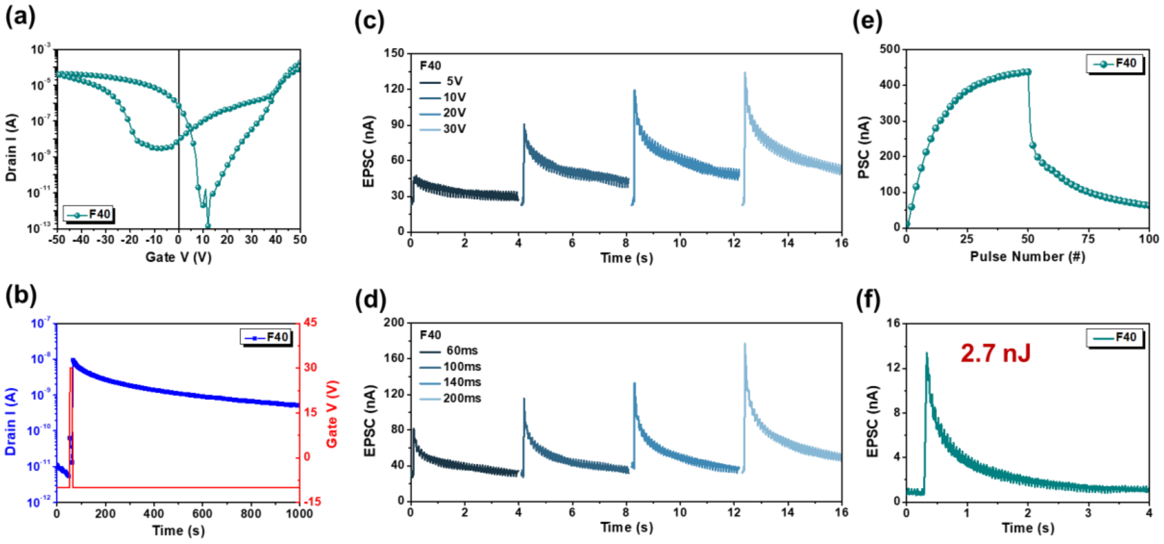

Figure S24. (a) Current-voltage hysteresis of the F40 transistor device using a 100 nm SiO_2 dielectric layer measured under a dual-sweep model. (b) Its charge retention time tests after biasing by a V_{GS} of 30 V for 3s. EPSC of this device triggered by (c) various gate pulse biases and (d) various gate pulse widths. (e) Current modulation of this device under presynaptic stimuli for electrical potentiation (50 pulse number, presynaptic spike voltage: +30 V, pulse width: 50 ms, and pulse interval: 30 ms, respectively) and habituation (50 pulse number, presynaptic spike voltage: −20 V, pulse width: 50 ms, and pulse interval: 30 ms, respectively). (f) EPSC of this device triggered by a single pulse with amplitude of +10 V and a width of 20 ms.

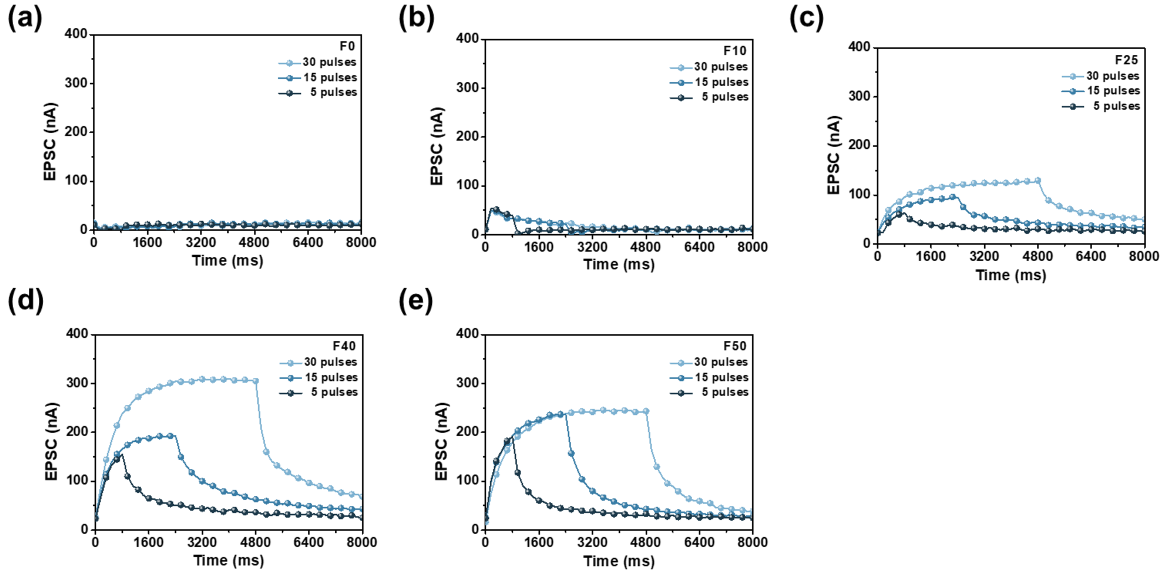

Figure S25. EPSC of the (a) F0, (b) F10, (c) F25, (d) F40, and (e) F50 devices triggered by 5, 15, 30 pulses (80 V for 100 ms per pulse and the pulse interval is 60 ms).

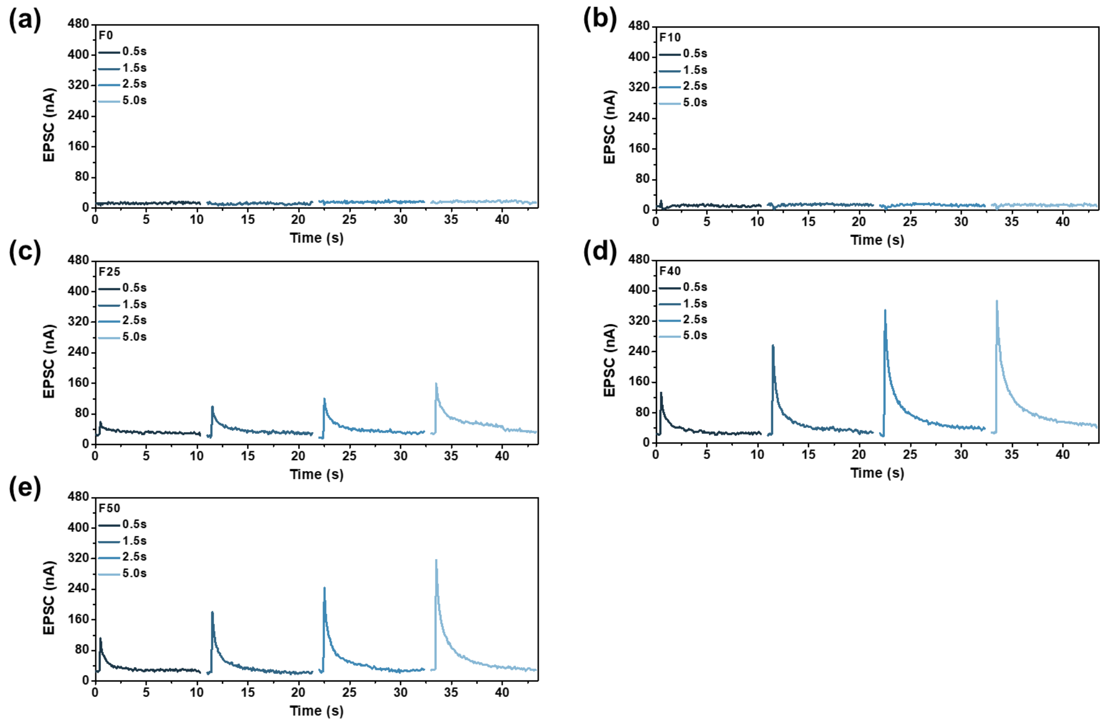

Figure S26. EPSC of the (a) F0, (b) F10, (c) F25, (d) F40, and (e) F50 devices triggered with various gate pulse widths.

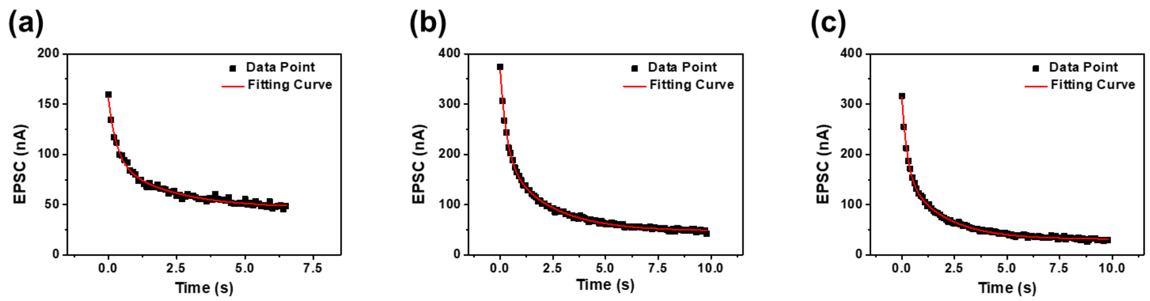

Figure S27. Fitting of the SDDP curves of the (a) F25, (b) F40, and (c) F50 devices triggered by a constant pulse (+80 V) with a pulse width of 5 s.

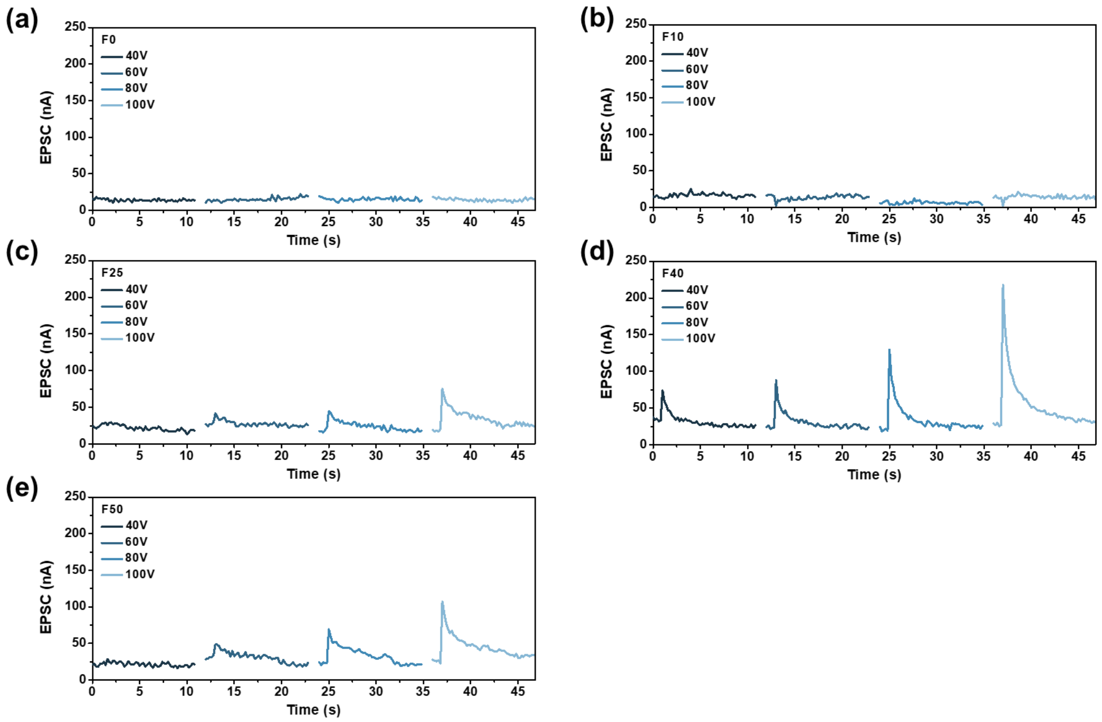

Figure S28. EPSC of the (a) F0, (b) F10, (c) F25, (d) F40, and (e) F50 devices triggered with various gate pulse biases.

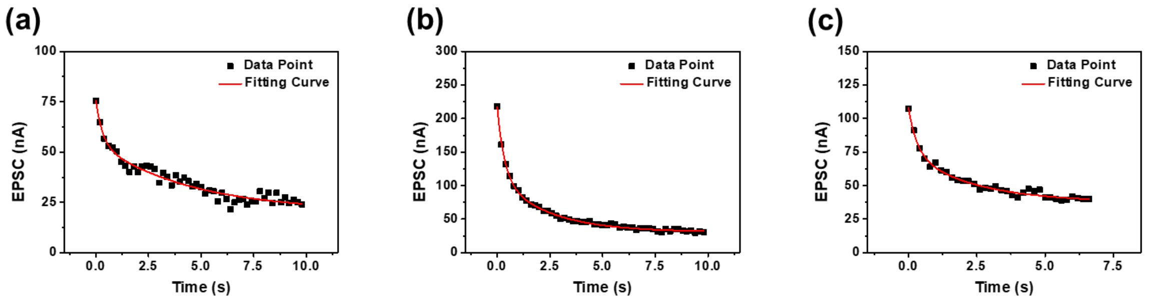

Figure S29. Fitting of the SVDP curves of the (a) F25, (b) F40, and (c) F50 devices measured at a constant gate bias of 100 V.

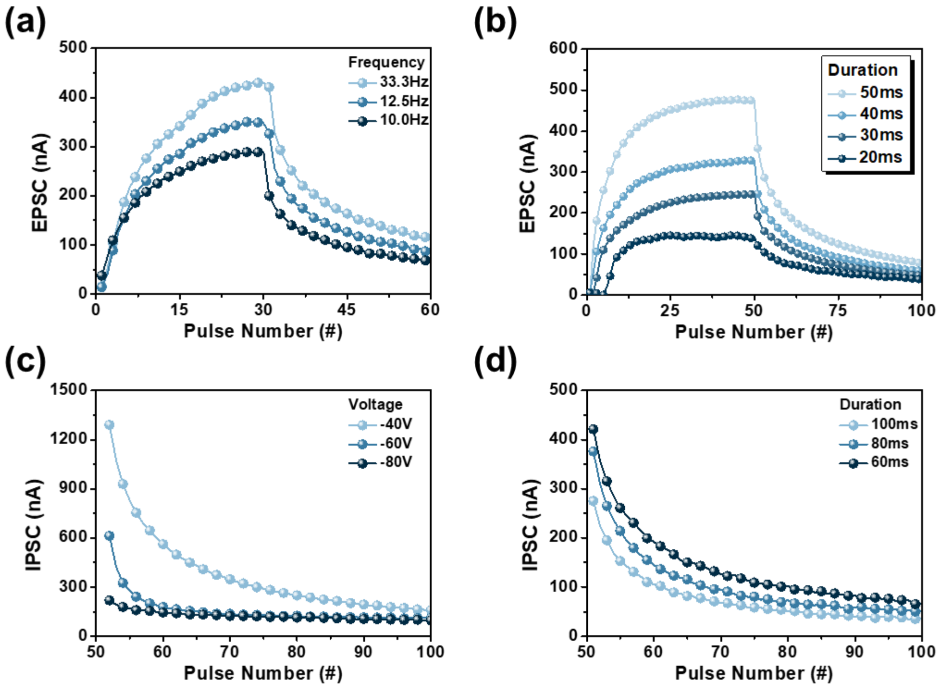

Figure S30. EPSC of the F40 device triggered by (a) 30 consecutive pulses with various frequencies and (b) 50 consecutive pulses with various pulse widths. IPSC of the F40 device triggered by (c) 50 consecutive pulses with various gate biases and by (d) 50 consecutive pulses with various pulse widths.
